# Adenovirus mediated gene therapy in cell lines derived from canine oral melanoma

**DOI:** 10.3389/fimmu.2026.1835389

**Published:** 2026-05-28

**Authors:** Otavio Augusto Rodrigues, Fernanda Antunes, Gissele Rolemberg Oliveira Silva, Bianca Naomi Niitsuma, Jean Carlos dos Santos da Luz, Bryan E. Strauss

**Affiliations:** Laboratório de Vetores Virais (LVV), Centro de Investigação Translacional em Oncologia (CTO)/LIM24, Instituto do Câncer do Estado de São Paulo (ICESP), Faculdade de Medicina, Universidade de São Paulo (FMUSP); Comprehensive Center for Precision Oncology (C2PO), Universidade de São Paulo, São Paulo, Brazil

**Keywords:** adenovirus, comparative oncology, gene therapy, interferon-β, p53/p14ARF pathway, translational research

## Abstract

The study of cancer in dogs can provide valuable insights into the basic biology and the development of novel cancer treatments as veterinary cases develop spontaneously, exhibit heterogeneity and closely match the biology of human cancers. Here we have isolated and characterized cell lines derived from spontaneous oral melanomas from companion dogs and subjected these cells to adenovirus-mediated gene transfer both *in vitro* and *in vivo*. Two newly established cell lines, BAN and TIG, were confirmed to be oncogenically transformed, as they bypassed G1 arrest under serum starvation and were tumorigenic in nude mice. These cell lines harbor wild-type p53, which, in response to treatment with doxorubicin or Nutlin-3, promoted the expression of well-known p53 target genes (CDKN1A, MDM2). The established cell lines are permissive to transduction with RGD-modified non-replicating adenoviral vectors and allowed reporter gene activity from p53-responsive promoter vectors controlled. Treatment with adenoviral vectors encoding canine p14^ARF^ and interferon-β (IFNβ) resulted in cell death with liberation of immunogenic cell death markers *in vitro* and reduction of tumor progression when subcutaneous tumors in nude mice were treated with *in situ* gene therapy. These results indicate that adenovirus-mediated delivery of p14ARF and IFNβ is effective in a canine model of oral melanoma, supporting the feasibility of applying comparative oncology approaches to the development of this gene therapy strategy.

## Introduction

Only 11% of anti-cancer therapies that show efficacy in mice will be approved for use in humans (1). The laboratory mouse fails to recapitulate various aspects of human clinical cases since the mice are genetically homogeneous, receive a controlled diet and living conditions, and studies are typically performed using animals of the same sex and age bearing transplanted tumors (1). Alternatively, comparative oncology, the study of naturally occurring cancers in animals as a model for human disease, may be critical for showing both efficacy and safety of novel therapeutic agents. Veterinary cases of cancer, such as oral melanoma, reflect the heterogeneity seen in human cases, including influence of environmental factors as well as spontaneous appearance and metastasis in the presence of the immune system (2–4). All model systems come with advantages and disadvantages and thus should be chosen to provide complementary information. Mouse models provide many opportunities, including the relative ease of xenograft models, extensive genomic data and the potential for genetic engineering (5), while canine cancers present the complexity of human cancers, including their etiology and diversity of the tumor microenvironment (2–4). Through clinical veterinary studies, relevant data can be generated while reducing the number of mice needed for translational research (2–4). A recent review highlights the testing of immunogene therapies in companion animals, concluding that such studies strongly support veterinary oncology and generate useful data for the pre-clinical assessment of future human trials (6).

Cancer is the cause of death in 40 to 50% of dogs over 10 years old and is responsible for 20 to 25% of deaths overall (7). Thus, as in humans, the occurrence of cancer in dogs is frequent and represents a considerable emotional and financial burden. Melanoma represents 7% of all tumors in dogs, with the oral cavity being the most prevalent location. The development of melanoma is more common in older dogs (10 to 11 years), with no tendency based on sex, but with elevated occurrence in certain breeds, including Golden Retriever and Rottweiler. Oral melanoma may be found in the gingiva, lips, tongue, tonsils, palate and oropharynx. Cases may present with a brown or black mass, though approximately one-third are amelanotic, and metastasis to the lungs is seen in 17 to 51% of cases. Treatment typically includes invasive surgery (mandibulectomy, maxillectomy) and while chemotherapeutics shows little benefit, tumors are often radiosensitive. With surgery, the one year survival rate for stage II – III cases is 20% and recurrence is seen in 22 to 48% of cases (8, 9). At the molecular level, canine oral melanomas share features of human mucosal melanoma, including mutation in *NRAS* or *KRAS* (10 to 20% of cases), *PTEN* (10%) and *TP53* (8 to 28%) and involvement of MAPK and PI3K pathways have been reported for canine oral melanomas (8–10). Similar to the human case, copy number variants of *MDM2* and deletion of *CDKN2A* have also been observed in canine oral melanoma (8–10). Alteration of *BRAF* is not frequently seen in canine oral melanomas (10). We point out that canine oral melanomas do not present mutation in SF3B1 or KIT (11), although these genes are altered in human mucosal melanoma (12), thus some differences are found when comparing species.

Our previous studies of gene therapy using a recombinant, non-replicating human adenovirus serotype 5 (Ad5) vector with modified tropism due to RGD (arginine-glycine-aspartic acid) tripeptide motif present in the fiber protein have focused on mouse and human melanoma cell lines (13, 14). Our approach targets both the p53/p14^ARF^ and type I interferon pathways to induce immunogenic cell death (15). In addition, we have shown that a p53-responsive promoter, which we call PG, provides high level transgene expression in the presence of functional p53 (15). While encouraged by our findings, the use of more clinically relevant models is needed to better represent the complexity of clinical cases of melanoma, including critical aspects of the immune response. We propose that the study of our approach using comparative oncology will reveal critical aspects of safety and efficacy. As a first step, here we isolated cell lines derived from canine oral melanomas and characterized their growth in nude mice, monolayer cultures, p53 status and response to transduction using adenoviral vectors encoding canine p14^ARF^ and IFNβ.

## Materials and methods

### Sample collection

The canine oral melanoma samples were obtained from spontaneous cases in companion pets treated at the Oncocane (São Paulo, SP, Brazil) and Dentalpet (São José dos Campos, SP, Brazil) veterinary clinics. The owners of the dogs signed an agreement for the surgical procedures in accordance with resolution No. 1071 of November 17, 2014, Article 4 III of the *Conselho Federal de Medicina Veterinária* as well as a consent form for collection of the samples and participation in this scientific study. The studies involving animal participants were reviewed and approved by the Committee for the Ethical Use of Animals (*Comissão de Ética no Uso de Animais*, CEUA), process 007/2016, Faculdade de Medicina Universidade de São Paulo (FMUSP).

### Histopathological evaluation of the clinical samples

Approximately half of each tumor was preserved in 10% formaldehyde, embedded in paraffin and sections stained with hematoxylin and eosin, melanoma cocktail (melan-A, HMB45, tyrosinase), cytokeratin cocktail (AE1, AE3) or PNL2 (performed by pathology services CEVAP, Adamantina, SP; PROVET, Sao Paulo, SP; VETPAT, Campinas, SP; or Vetmol, Botucatu, SP, Brazil).

### Tumor dissociation and growth of primary cells in monolayer culture

The remaining portion of the fresh tumor was transported in RPMI supplemented with 1x antibiotic-antimycotic reagent (both from Thermo Fisher Scientific, Waltham, MA, USA) and kept on ice until processing. Tumor fragments were minced with a sterile scalpel, washed in 1x PBS then digested with 0.25% trypsin (Thermo Fisher Scientific) at 37°C, 30 m, continuous agitation. Trypsin was quenched by addition of RPMI containing 10% fetal bovine serum (FBS, Thermo Fisher Scientific), then a 1% liberase solution (Sigma-Aldrich, St. Louis, MO, USA) was added (8 µl/100 mg of tumor) before incubation at 37°C, 30 m, with continuous agitation. Cells were then washed with RPMI/10% FBS before transfer to 6-well dishes and cultivation in the presence of RPMI/10% FBS/1x antibiotic-antimycotic at 37°C in a humidified incubator, 5% CO_2_ atmosphere. The mixed cell populations were cultivated for 2–3 weeks with periodic sub cultivation as needed. Cells were then collected, counted and a solution containing 1 cell/100 µl of medium (RPMI/10% FBS/1x antibiotic-antimycotic) was distributed (100 µl/well) in 96 well dishes. Wells with a single colony were trypsinized and the cellular clones expanded. Cells were monitored for *Mycoplasma* sp. using a PCR assay. The resulting BAN and GAB primary cultures were expanded and cryopreserved.

### Growth kinetics (monolayer)

Cells were seeded in 6-well dishes, 5x10^4^ cells/well, and cultivated as described above. At 24, 48 and 72 h, cells were harvested and viable cells counted manually (trypan blue exclusion). The doubling time was calculated using the formula: Doubling time = duration x ln (2)/ln(final concentration/initial concentration) and using on online resource (https://www.omnicalculator.com/biology/cell-doubling-time).

### Serum starvation

Cells were seeded at 1x10^5^ cells/well in 6-well dishes. The next day, the medium was replaced with fresh medium containing 10%, 2% or 0.5% FBS. The cells were then incubated at 37 ˚C until capturing photomicrographs (EVOS FL, Thermo Fisher Scientific) and harvesting of duplicate wells for each condition. The cells of each replicate were fixed in 70% ethanol, then treated with RNAse and stained with propidium iodide (PI). Flow cytometry was performed (Attune, Thermo Fisher Scientific) and analyzed (FlowJo, Becton, Dickinson & Company, East Rutherford, NJ, USA).

### Tumor formation in nude mice

The studies involving mice were reviewed and approved by the CEUA-FMUSP, protocol numbers 007/2016 and 1088/2018. Balb/c nude mice, female, 6 to 8 weeks of age, were provided by the Biotério Central (FMUSP) and all procedures were performed at the Centro de Medicina Nuclear (CMN, FMUSP). To quantify tumor growth, 1x10^6^ cells of the indicated cell lines were injected s.c. while under anesthesia (isoflurane, 4%, inhaled). The animals were monitored and tumor growth was assessed using a digital caliper. Tumor volumes were determined as per Steel (1977) (16); V=Dxd^2^/2 (V, volume; D, larger diameter, d, smaller diameter). However, these animals also received four i.t. injections of PBS, 50 μl each occasion, where the first was applied when the tumor volume was 60 mm^3^ and repeated at 48-hour intervals. In the case of BAN, these same animals were used as controls for the *in situ* gene therapy assay.

### Sequencing of exons 4–8 of *TP53*

The established cell lines were used as the source for total RNA isolation using TRIzol (Thermo Fisher Scientific) as per the manufacturer’s instructions. The quantity of recovered RNA was assessed using A260 measurements (Nanodrop, Thermo Fisher Scientific), while purity was evaluated by the A260/A280 ratio, and integrity was confirmed through visualization of 28S and 18S ribosomal RNA bands by gel electrophoresis. Conventional RT-PCR was performed using the SuperScript One-Step RT-PCR System (Thermo Fisher Scientific) and primers F4 (5’ TACTCCCCTCTCCTCAACAAG 3’), F6 (5’ GTTGGCTCTGACTATACCACC 3’) and R8 (5’ CTGAAGGGTGAAATATTCTCCA 3’). The amplification products were sequenced using the BigDye Terminator v3.1 kit (Thermo Fisher Scientific) and the F4 and R8 primers. The reaction products were precipitated, washed and then evaluated using an ABI 3500 Genetic Analyzer (Thermo Fisher Scientific). Data was analyzed using DNASTAR (Madison, WI, USA) and compared to known sequences for canine *TP53* (NC006587.3 and AF060514.1) with the aid of BLAST (NCBI, Bethesda, MD, USA).

### Adenoviral vectors

AdRGD-PG-eGFP, AdRGD-PG-Luc2 and AdRGD-CMV-Luc2 were constructed in our laboratory and have been previously reported (13, 14, 17). A synthetic cassette encoding the p14^ARF^ and IFNβ cDNAs was designed based on the canine standard DNA sequences from GeneBank [interferon-β (FJ194477.1) and p14^Arf^ (FM883643.1)]. Cassette synthesis was performed by GeneArt Gene Synthesis (Therma Fisher Scientific). To facilitate the detection of the canine proteins by western blotting and/or immunofluorescence, tag sequences were placed downstream of p14^ARF^ (FLAG-tag: DYKDDDDK) and downstream of IFNβ (c-myc tag: EQKLISEEDL). The inclusion of strategically placed restriction enzyme sites allowed for the insertion of the full-length cassette in a pEntr vector that also encoded the p53 responsive PG promoter (pEntr-PG). The restriction sites also allowed for the removal of components as desired from the pEntr-PG constructs, resulting in the pEntrPGcp14Flag and pEntrPGcIFNbmyc vectors. Adenoviral constructs were generated by site-specific recombination between the ‘pEntr’ vectors and the ‘pDest’ vector encoding the adenoviral backbone in the presence of Clonase LR (Thermo Fisher Scientific) as previously reported (13, 14, 17). Afterwards, the desired clones were identified, thus generating the AdRGD-PG-cp14^ARF^ and AdRGD-PG-cIFNβ vectors (see **Supplementary Figure 4** for vector maps). Vectors were produced by iodixanol gradient centrifugation as per published protocols (13, 14, 18) and titration was performed using the Adeno-X Rapid titration kit (Takara Bio). This biological titer, expressed as transducing units (TU)/ml, was used in calculating the multiplicity of infection (MOI) for each assay.

### Transduction with adenovirus and drug treatments

Cells, 1x10^5^ cells/well unless otherwise noted, were mixed with the appropriate amount of virus and seeded into 6-well plates in DMEM containing 5% FBS. The UACC62 cell line was kindly provided by Dr. Roger Chammas (Instituto do Câncer do Estado de São Paulo) and verified by STR analysis. The cells were incubated, 37°C as indicated in the experiment. When used, doxorubicin (0.1 µM final concentration, Sigma-Aldrich) or Nutlin-3 (10 µM final concentration, Sigma-Aldrich) was added 24 h after transduction and then the cells were incubated, 37°C, for the time indicated in the assays. For the reporter assays, a MOI of 25 was used. However, assays using the p14ARF and IFNβ vectors were performed at a higher MOI (400) to accentuate the impact of the therapeutic gene. To maintain consistent MOI and gene dosage, individual transduction with p14^ARF^ or IFNβ (MOI 200) was complimented with GFP-encoding vector (MOI 200). For the GFP control, a total MOI of 400 was used. Thus, whether administered individually or in combination, gene transfers were performed at a constant total MOI of 400, with the dosage of the p14ARF or IFNβ vectors remaining unchanged.

### Immunofluorescence assay

For each condition, a 12 mm round coverslip was placed in the well of a 24-well tissue culture dish. Fifty-thousand cells were mixed with the indicated virus in DMEM 5%FBS, MOI = 200/vector, and plated. Note that the GFP vector was not mixed with p14ARF or IFNβ to avoid background fluorescence. Before detection of IFNβ, cells were incubated in the presence of brefeldin-A, 5μg/mL, for 4h, to promote intracellular accumulation of the protein. After 48 hours of incubation at 37 °C, the cells were fixed with 4% paraformaldehyde for 10 minutes at room temperature, followed by three washes with Tris-buffered saline (TBS). Then the cells were permeabilized with 0.3% Triton X-100 in TBS for p14ARF labelling and 0.1% Triton X-100 in TBS for IFNβ labelling and washed 3 times with TBS-0.05% Tween 20 (TBST). Blocking was performed using 5% bovine serum albumin (BSA) in TBS, incubated for 30 min at r.t. Then the primary antibody was added (mouse anti-flag 1:200 dilution, Cat. MA1-91878, Invitrogen or mouse anti-myc 1:200 dilution, Cat. MA1-21316, Invitrogen), incubated for 1.5h, r.t, before washing 3 times with TBST. The secondary antibody was goat anti-mouse Alexa Fluor 555 (1:500 dilution, Cat. A-21422, Invitrogen) diluted in TBST-BSA 1%, added to the wells and incubated for 1.5h, r.t, before washing 3 times with TBST. Then, nuclear staining was performed using Hoechst (1:5000 in TBS), 15 min incubation at r.t. before a final round washing with TBST. Cover slips were mounted on glass microscope slides with 3 µl of mounting medium (Fluoromount-G, Cat. 00-4958-02, Thermo Fisher). Fluorescence microscopy and photo documentation were performed using a Leica DMi8, 40x objective. Scale bar included in each photomicrograph = 100 µm.

### Detection of p53 by flow cytometry

Cells were seeded at 5 × 10^4^ cells per well in 12-well plates and treated the following day with doxorubicin (0.1 µM final concentration, Sigma-Aldrich) or Nutlin-3 (10 µM final concentration, Sigma-Aldrich), followed by incubation at 37 °C for 36 h. Cells were then harvested and fixed with 4% paraformaldehyde (PFA) for 15 min at room temperature, followed by permeabilization with 100% methanol at -20°C for 10 min. After blocking with PBS containing 1% BSA, cells were incubated with anti-p53 antibody (1:100; sc-126, Santa Cruz Biotechnology) for 1 h at room temperature. After washing with PBS-1% BSA, cells were incubated with an anti-mouse Alexa Fluor 488 secondary antibody (1:1000 - A11001, Invitrogen) for 30 min at room temperature. Cells were washed with PBS and analyzed by flow cytometry using an Attune cytometer (Thermo Fisher Scientific). Data analysis was performed using the geometric mean fluorescence intensity (MFI) from the 488 nm channel with FlowJo (Becton, Dickinson & Company, East Rutherford, NJ, USA).

### RT-qPCR

Cells were plated as described above and, when indicated, treated with doxorubicin or Nutlin-3, incubated 48 h at 37 ˚C. Alternatively, the cells were transduced using the vectors and MOI indicated in the figure and incubated for 48h at 37 °C. Then total RNA was isolated using the TRIzol reagent (Thermo Fisher Scientific) according to the manufacturer’s instructions. The PCR assays were performed using GoTaq^®^ 1-Step RT-qPCR System (Promega) and 7500 Real-Time PCR System (Thermo Fisher Scientific) as recommended by the supplier. Expression levels were analyzed using the ΔΔCt method. For the canine cell lines, HPRT1 was used as the reference gene whereas GAPDH was used for the human cell line. Oligonucleotide sequences are presented in **Supplementary Table 3**.

### Detection of eGFP

Cells were trypsinized, washed, and resuspended in 1x PBS before flow cytometry (Attune, Thermo Fisher Scientific). The percentage of eGFP-positive cells and the mean intensity of eGFP activity were determined using FlowJo software (Becton, Dickinson & Company).

### Luciferase assay

Cell lysates and luciferase reactions were prepared as per the instructions of the Luciferase Assays System (Promega, Madison, WI, USA). Luciferase activity was observed using a Victor3–1420 Multilabel Counter (Perkin Elmer, Waltham, MA, USA) and values normalized to protein content of the lysates, determined using a standard Bradford assay.

### MTT assay

Cells were seeded in 96 well dishes, 2.5x10^3^ cells/well, and triplicate wells were transduced the following day with the vector and MOI as indicated in the assay. At the end of the indicated incubation period, medium was replaced for RPMI/10% FBS containing 5 mg/mL MTT (Sigma-Aldrich) and the plates were incubated at 37°C for 4 h. To each well, 100 µl of 20% SDS, 50% DMF in water, pH 4.7, was added. The dishes were protected from light and incubated at 37°C for 16 h. The result was measured at 570 nm using the Victor3–1420 Multilabel Counter (Perkin Elmer).

### ICD markers

Fifty-thousand cells were mixed with the indicated virus in DMEM 5% FBS, MOI = 200/vector, and plated on 12 wells dish. After 36hs, supernatants were collected and ENLITEN™ ATP Assay System reagent (Promega) was used to reveal the release of ATP into the supernatant and was detected using the GloMax^®^ Explorer SystemGM3510 (Promega). Detection of HMGB1 released into the supernatant was performed by first concentrating the supernatant, then western blotting with anti-human-HMGB1 antibody (1:1000, ab79823; ABCAM, Cambridge, UK) and anti-Rabbit -HRP secondary antibody (1:1000, A0545 – Sigma-Aldrich). Detection was performed using ECL Prime Western Blotting Detection Reagents (Cytiva, Uppsala, Sweden) and ImageQuant LASS 4000 photodocumentation system. For Calreticulin analysis both the supernatant and cells were collected followed by anti-CalR (1:500 - ab2907; ABCAM, Cambridge, UK) incubation for 1h at 4°C. After washing with PBS-1% BSA, cells were incubated with an anti-rabbit Alexa Fluor 488 secondary antibody (1:1000 - A21206, Invitrogen) for 30 min at 4°C. Cells were washed with PBS and analyzed by flow cytometry using an Attune cytometer (Thermo Fisher Scientific). Data analysis was performed using the geometric mean fluorescence intensity (MFI) in the 488 nm channel with FlowJo (Becton, Dickinson & Company, East Rutherford, NJ, USA).

### *In situ* gene therapy

For the *in situ* gene therapy assay, 1x10^6^ BAN cells resuspended in 100 μL of 1x PBS were inoculated in the left flank of the mice. After 38 days, when the tumor volume was between 15 and 50 mm^3^, mice were treated with four intratumor injections of a total 1×10^9^ infectious units of AdRGD-PG vectors (eGFP alone, p14^ARF^ + eGFP, IFNβ + eGFP, or p14^ARF^ + IFNβ, thus maintaining an equal vector and gene dosage among all conditions), 50 μl total volume, at 48 h intervals. Tumor volume was determined by measurement with a digital caliper. Mice were euthanized (anesthesia with isoflurane, 4%, inhaled, followed by CO_2_ inhalation) when tumor volume reached no more than 1000 mm^3^, animal weight loss of around 4% of body mass occurred or whose tumors showed signs of exposed necrosis.

### Statistical analysis

Statistical analysis was performed using the Prism 9 software (GraphPad, Boston, MA, USA). Analyses used in each experiment are indicated in each figure’s legend. Data was considered significant when p<0.05.

## Results

### Isolation and characterization of canine oral melanoma cell lines

Tumor samples were obtained during biopsy or surgical resection of oral melanomas from companion dogs. A portion of the tumor was subjected to histopathology (**Table 1**), revealing that all cases were indeed melanoma, though 5/9 were amelanotic. The remaining portion was transported to the laboratory where mechanical and enzymatic dissociation was performed. The mixed cell populations were cultivated as monolayers before cellular clones were isolated. We then tested each clone for tumor formation in nude mice (data not shown). The tumors were recovered and subjected to histopathological characterization (**Supplementary Table 1**). A portion of these same tumors was dissociated and then cultivated as a monolayer. The BAN (clone C10) and TIG (clone G2) cell lines (from patients 3 and 1, respectively) were expanded, cryopreserved and used in the remaining assays. STR profiles of the cell lines as well as genetic confirmation of the breeds are shown in **Supplementary Table 2**.

**Table 1:** Clinical characteristics of donors and oral melanoma samples.

Dog				Tumor				
N°	Breed	Age	Sex	Location	Stage	Stage	Histologic presentation	Diagnosis
1	German shepherd	10	M	Soft palate	T3N1M1	IV	Mixed	Amelanotic melanoma
2	Poodle	15	M	Mandible	T3N0M0	III	Fusiform	Melanoma
3	Akita	13	M	Maxilla	T3N0M0	III	Mixed	Amelanotic melanoma
4	Golden retriever	1	F	Maxilla	T3N0M0	III	Fusiform	Amelanotic melanoma
5	Mixed	15	F	Soft palate	T2N1M1	IV	Epithelioid	Melanoma
6	Poodle	11	M	Maxilla	T3N0M0	III	Epithelioid	Melanoma
7	Rottweiler	12	F	Soft palate	T3N0M0	III	Fusiform	Melanoma
8	Mixed	7	M	Mandible	T3N1M0	III	Fusiform	Amelanotic melanoma
9	Mixed	12	M	Superficial cervical lymph node	T3N1M1	IV	Epithelioid	Amelanotic melanoma

**Figure 1:**
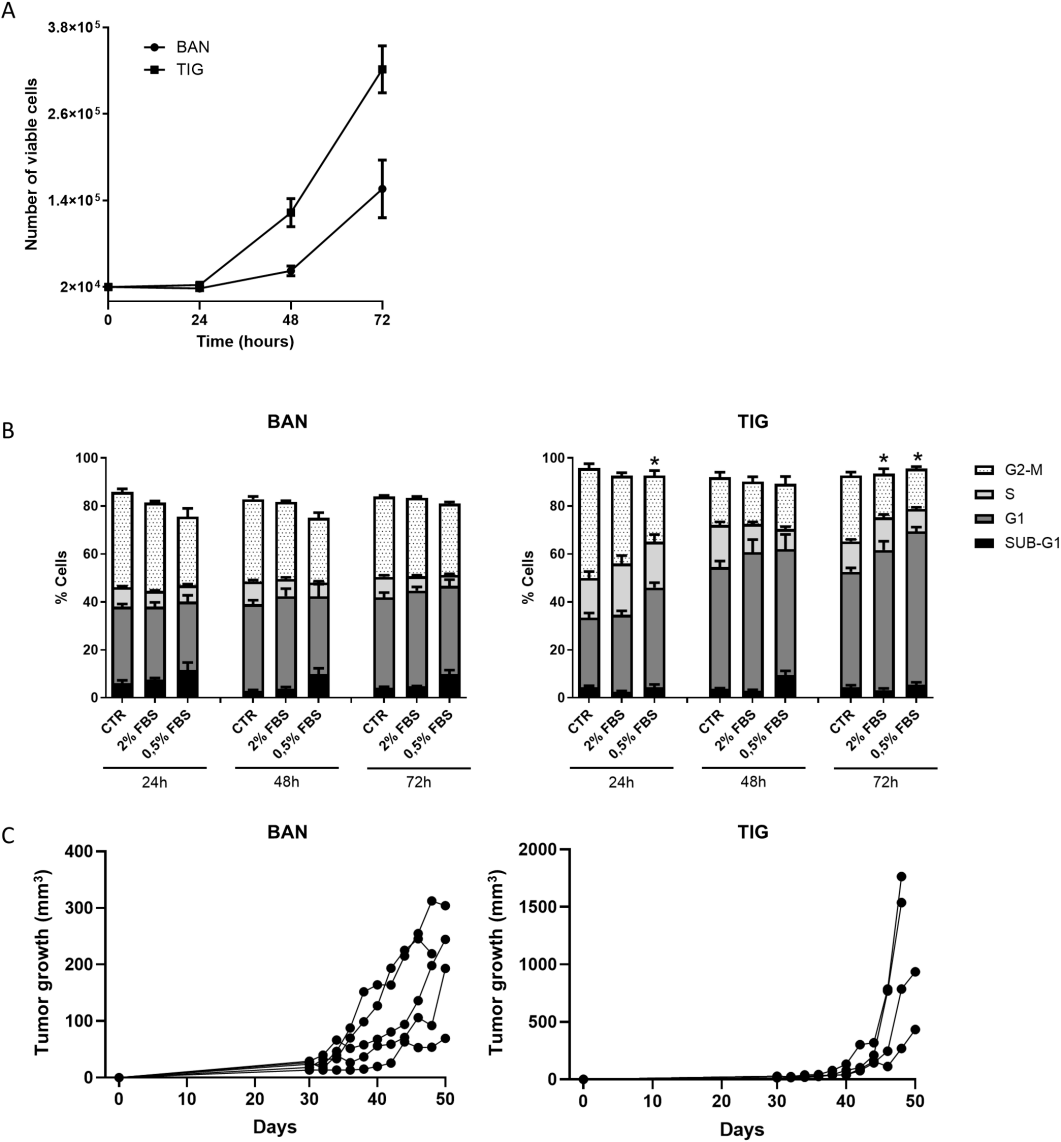
Growth of established canine melanoma cell lines. **(A)** the cell lines were plated (2x10^4^ cells/well in 24-well dishes) and cultivated for the indicated time before counting viable cells. The growth kinetics of each cell line are shown by the mean ± standard deviation from three independent biological assays, each performed with technical duplicates. **(B)** the BAN and TIG cell lines were cultivated in the presence of 10%, 2% or 0.5% FBS for the indicated times before being collected, fixed, stained with PI and the cell cycle distribution observed by flow cytometry. The graphs represent the mean ± standard deviation from three independent biological assays, each performed with technical duplicates. Only G1 was considered for statistical analysis. *p<0.05. Two-way ANOVA with Bonferroni statistical correction. **(C)** tumor growth in nude mice of established cell lines. For each cell line, 1x10^6^ cells were injected s.c. in nude mice and tumor volume was monitored over time. N = 5 for BAN; N = 4 for TIG.

We performed a series of assays to examine the proliferation and transformed phenotype of the cell lines. The growth kinetics of the cell lines were observed (**Figure 1A**) and the doubling times were 22h42m and 18h0m for BAN and TIG, respectively. We next explored the response of the cell lines to serum starvation where the cells were cultured in 10%, 2% or 0.5% serum before capturing photomicrographs (**Supplementary Figure 1**) and examining the cell cycle (**Figure 1B**; **Supplementary Figure 2**). While neither of the cell lines exhibited overt G1 arrest, a statistically significant accumulation of G1 cells was seen only for the TIG cell line. The kinetics of s.c. tumor formation in nude mice were also measured starting with the injection of tumor cells on day 0 (**Figure 1C**), revealing that BAN progressed more slowly than TIG. These assays suggest that the cell lines established in this work are indeed transformed and tumorigenic.

**Figure 2:**
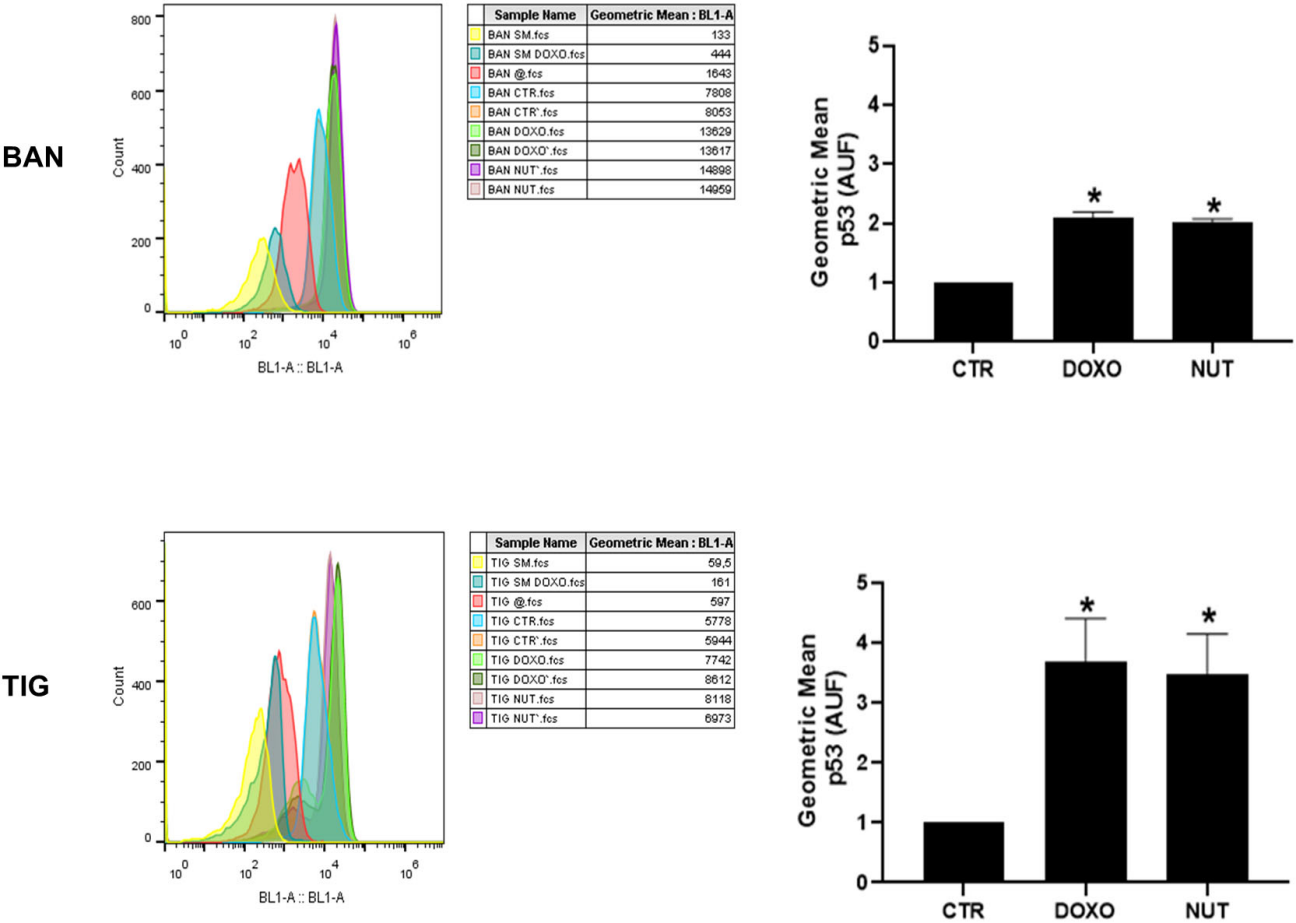
Detection of p53 protein by flow cytometry. The BAN and TIG cell lines were cultivated without treatment (CTR) or subjected to treatment with doxorubicin (DOXO) or Nutlin-3 (NUT) for 36 hours before harvesting, fixing, permeabilizing and staining for p53. The flow cytometry data (representative example shown) was analyzed and the geometric mean used to calculate relative p53 levels. AUF, arbitrary units of fluorescence, normalized to the CTR condition. The assay was performed on at least three independent occasions with each condition performed with experimental duplicates. *p<0.05 – one-way ANOVA with Dunnett’s post test.

We were especially interested in determining the status of *TP53* in the newly established cell lines. Sequencing *TP53* exons 4 to 8, the region most frequently subject to mutation, revealed no alterations compared to the reference canine *TP53* sequence (**Supplementary Figure 3**). Since detection of the p53 protein was not observed by immunohistochemistry performed on tumor sections (**Supplementary Table 1**), we reasoned that treatment with a DNA damaging agent (doxorubicin) or an inhibitor of p53-MDM2 interaction (Nutlin-3) may be necessary to activate and stabilize p53. As seen in **Figure 2**, p53 protein was detected by flow cytometry in cells that were treated with p53-activating drugs.

**Figure 3:**
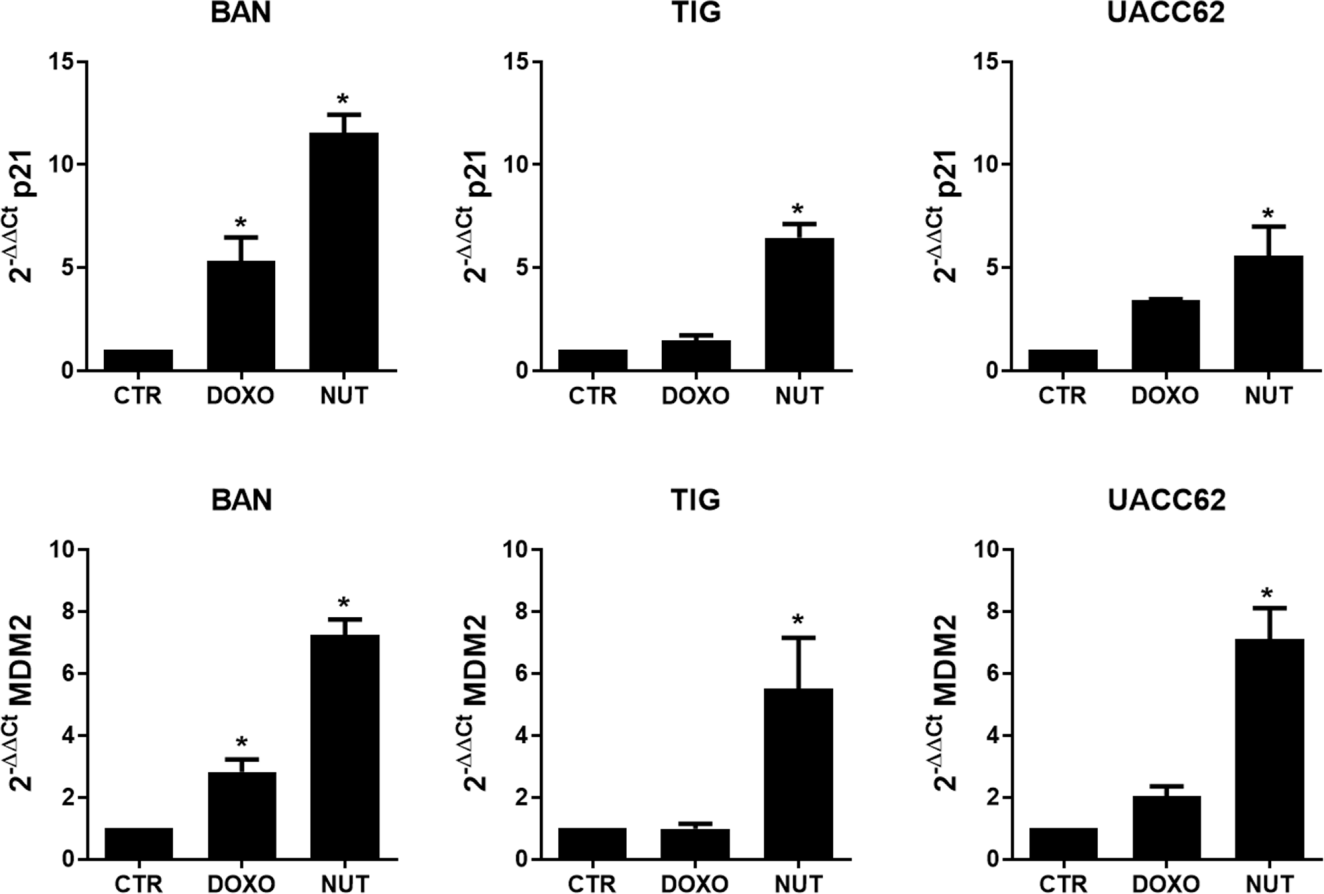
Upregulation of p53 target genes measured by RT-qPCR. The cell lines were plated and, the next day, treated with 0.1 μM doxorubicin (DOXO) or 10 μM Nutlin-3 (NUT) then incubated for 48 hours before extraction of total RNA. RT-qPCR was performed where GAPDH served as an internal control. The data represents three biological assays where each condition was performed in duplicate. *p<0.05 – one-way ANOVA with Dunnett’s post test.

We also examined the transcript levels of p53 target genes with and without drug treatment. As seen in **Figure 3**, the p21 (CDKN1A) and MDM2 transcripts, as measured by RT-qPCR, were elevated in response to drug treatment, especially Nutlin-3. The human melanoma cell line, UACC62, known to harbor wild type p53 (17, 19), was included as a positive control. These results suggest that endogenous p53 in the canine melanoma cell lines is functional.

### Transduction of the canine oral melanoma cell lines with adenoviral vectors

Before using these cell lines as models of adenovirus-mediated gene therapy, we tested their susceptibility to transduction using RGD-modified, non-replicating, E1/E3-deleted, adenovirus serotype 5 vectors (**Supplementary Figure 4**). Using a multiplicity of infection (MOI) of 25, transduction of approximately 30% of BAN and 50% of TIG was seen. Higher MOIs resulted in increased transduction efficiency but were also associated with toxicity, as evidenced by the accumulation of Sub-G1 (hypodiploid) cells (**Supplementary Figures 5**, **6**). When using our adenoviral vector containing a p53-responsive promoter (PG) to drive eGFP expression, it is possible that drug treatment would stimulate p53, resulting in increased eGFP activity. As seen in **Supplementary Figure 7**, the intensity of eGFP activity was elevated when cells transduced with AdRGD-PG-eGFP were treated with doxorubicin and Nutlin-3. Consistent with the notion mentioned above, increased p53 activity appears to drive higher levels of transgene expression, facilitating the detection of reporter activity in the transduced cells.

**Figure 4:**
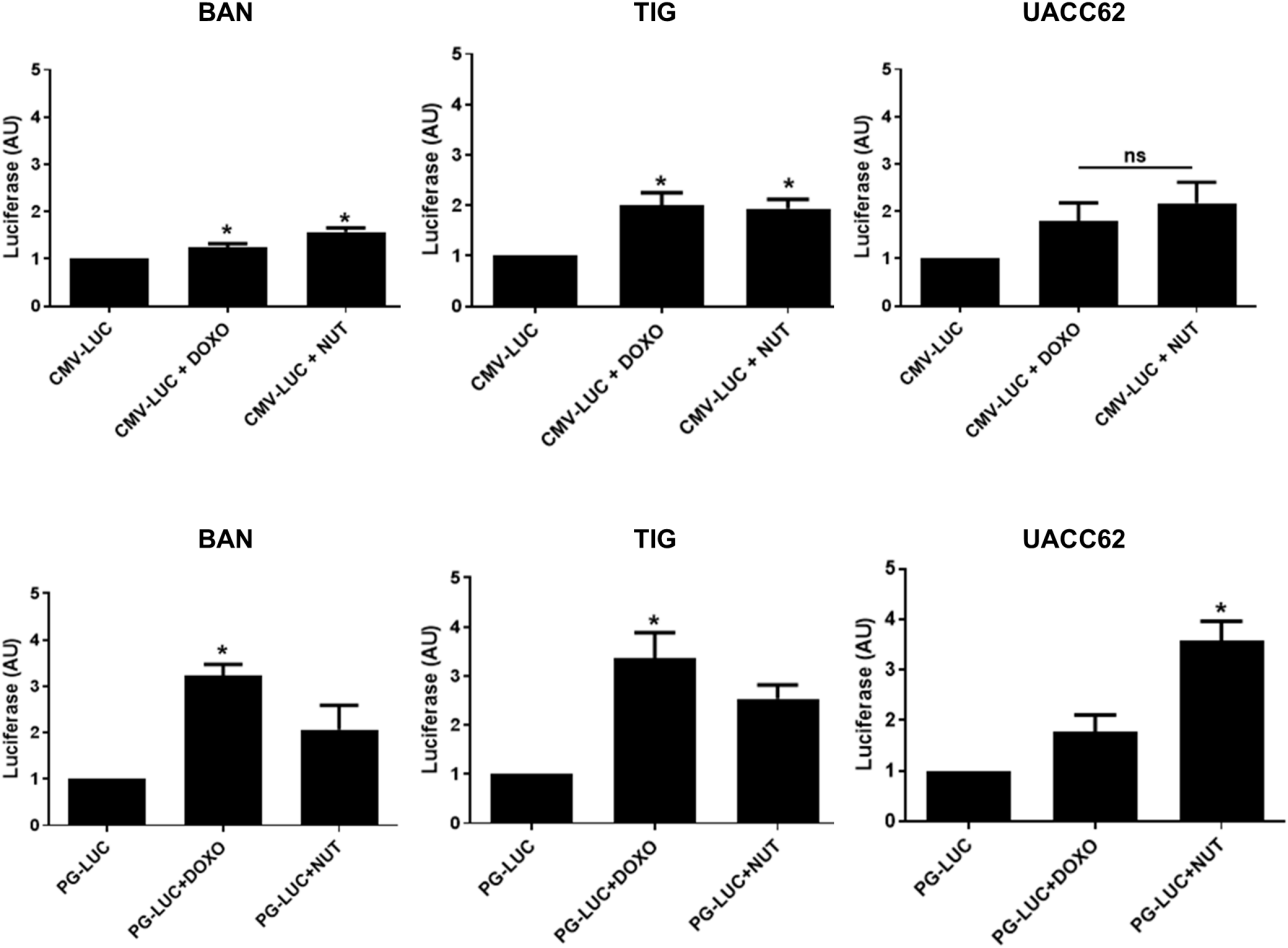
Reporter activity elevated in a p53-dependent manner. The cell lines were transduced (MOI 25) with either the constitutive AdRGD-CMV-Luc2 (CMV-LUC) or the p53-responsive AdRGD-PG-Luc2 (PG-LUC) vectors. The next day, the cells were treated with 0.1 μM doxorubicin (DOXO) or 10 μM Nutlin-3 (NUT) then incubated for 48 hours before harvesting for the luciferase assay. Luciferase activity (arbitrary units, a.u.) was normalized to protein content in the cell lysate then values were normalized against the vector control (CMV-LUC or PG-LUC). Graphs represent the mean and standard deviation from three biological assays with each condition in duplicate. *p<0.05 – one-way ANOVA with Dunnet post test. ns, not statistically significant.

We next tested whether the increase in reporter activity in response to drug treatment was dependent on the p53-responsive promoter driving transgene expression. To this end, the cell lines were transduced with adenoviral vectors encoding luciferase under the control of either the constitutive CMV promoter or the PG promoter, with the expectation that p53 activation would preferentially enhance expression from the p53-responsive PG promoter. **Figure 4** shows that doxorubicin and Nutlin-3 treatment increased reporter activity in the p53-responsive vector to a greater extent than seen with the CMV promoter. The human UACC62 cell line was included as a control.

### Using the BAN canine oral melanoma cell line as a model for p14^ARF^ and IFNβ gene therapy

Having established that the BAN and TIG cell lines are permissible to transduction with adenovirus and that the endogenous p53 could drive expression from the PG promoter, we next tested our gene therapy approach. For this, adenoviral vectors were constructed encoding the canine p14^ARF^ or canine interferon-β (IFNβ) under the control of the p53-responsive promoter (PG) (please see **Supplementary Figure 4** for vector details). Expression of the p14^ARF^ and IFNβ transgenes was confirmed by immunofluorescence, as shown in **Supplementary Figure 8**.

**Figure 5:**
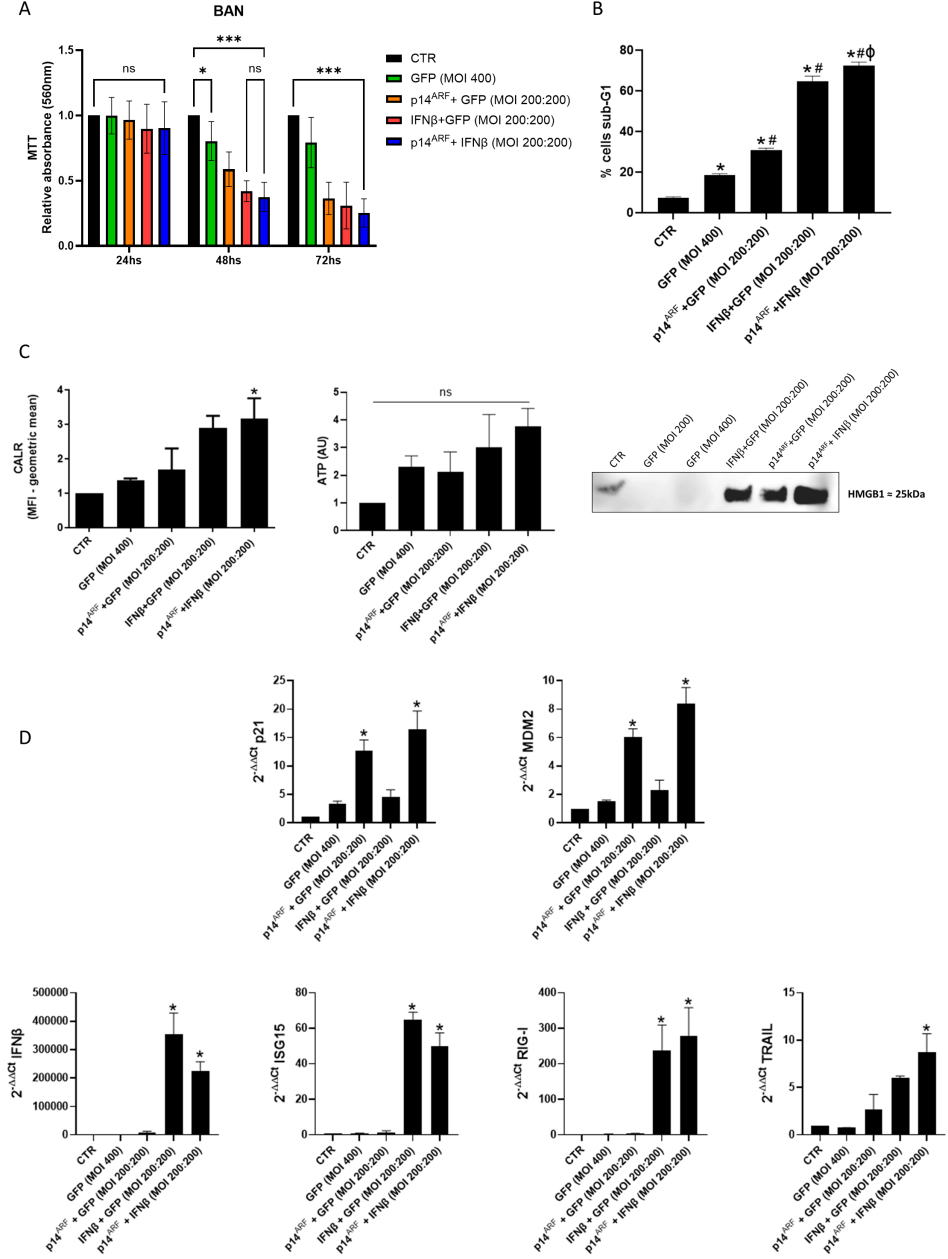
Response of BAN to *in vitro* treatment with p14ARF and IFNβ. Cells were transduced with consistent MOI (400) and gene dosage, as indicated. **(A)** MTT assay where cells were transduced in 96 well plates, stained and evaluated at the indicated time points. **(B)** sub-G1 assay where cells were transduced in 6-well plates, incubated for 72 hours before harvesting, fixation, and staining with PI. N = 4 biologic assays each performed with technical duplicates. *p<0.05 vs CTR; ***p<0.001 vs CTR; ^#^p<0.05 vs. GFP; ^φ^p<0.05 vs p14^ARF^+GFP and IFNβ+GFP. Ordinary one-way ANOVA, Bonferroni’s post test. **(C)** immunogenic cell death markers were examined 36 hours post-transduction. Calreticulin (CALR), fresh cells were stained and examined by flow cytometry. ATP, supernatant was collected and tested using a luminescence assay. The signal from the non-treated control (CTR) was considered as 1 and used to normalize the signal from the other conditions. AU, arbitrary units. HMGB1 was detected by western blotting using supernatants from the cultured cells. **(D)** RT-qPCR was performed using total RNA isolated from the cells 48 hs post transduction. **(A, C, D)**, at least 3 biological assays were performed. *p<0.05; 2-way ANOVA, Tukey’s post test. ns, not statistically significant.

Transduction of the BAN cell line resulted in reduced cellular viability, as demonstrated by a reduction of MTT activity (**Figure 5A**) and the accumulation of sub-G1 (hypodiploid) cells (**Figure 5B**; **Supplementary Figure 9**), especially when the treatment included IFNβ. The immunogenic cell death marker calreticulin was significantly increased only by the combined treatment (**Figure 5C**). At least qualitatively, both ATP and HMGB1 accumulated in the culture supernatant to a greater extent when the combined gene therapy was applied. P14^ARF^ gene transfer, alone or in combination with IFNβ, was associated with elevated p21 (CDKN1A) and MDM2 transcript levels (**Figure 5D**), showing activation of key p53-target genes. In the case of IFNβ gene transfer, we observed elevated levels of critical factors in the type I interferon pathway, such as ISG15, RIG-1 and TRAIL. Note that p14^ARF^ gene transfer alone was sufficient to alter cell viability, induce HMGB1 and elevate TRAIL expression. Together, these assays suggest that combined p14^ARF^ and IFNβ gene therapy was especially effective for the induction of cell death associated with the detection of ICD markers and activation of the p53 and type I IFN pathways in the BAN cell line. For TIG, accumulation of sub-G1 cells and activation of the p53 and type I IFN pathways was also evident upon gene transfer (**Supplementary Figure 10**).

**Figure 6:**
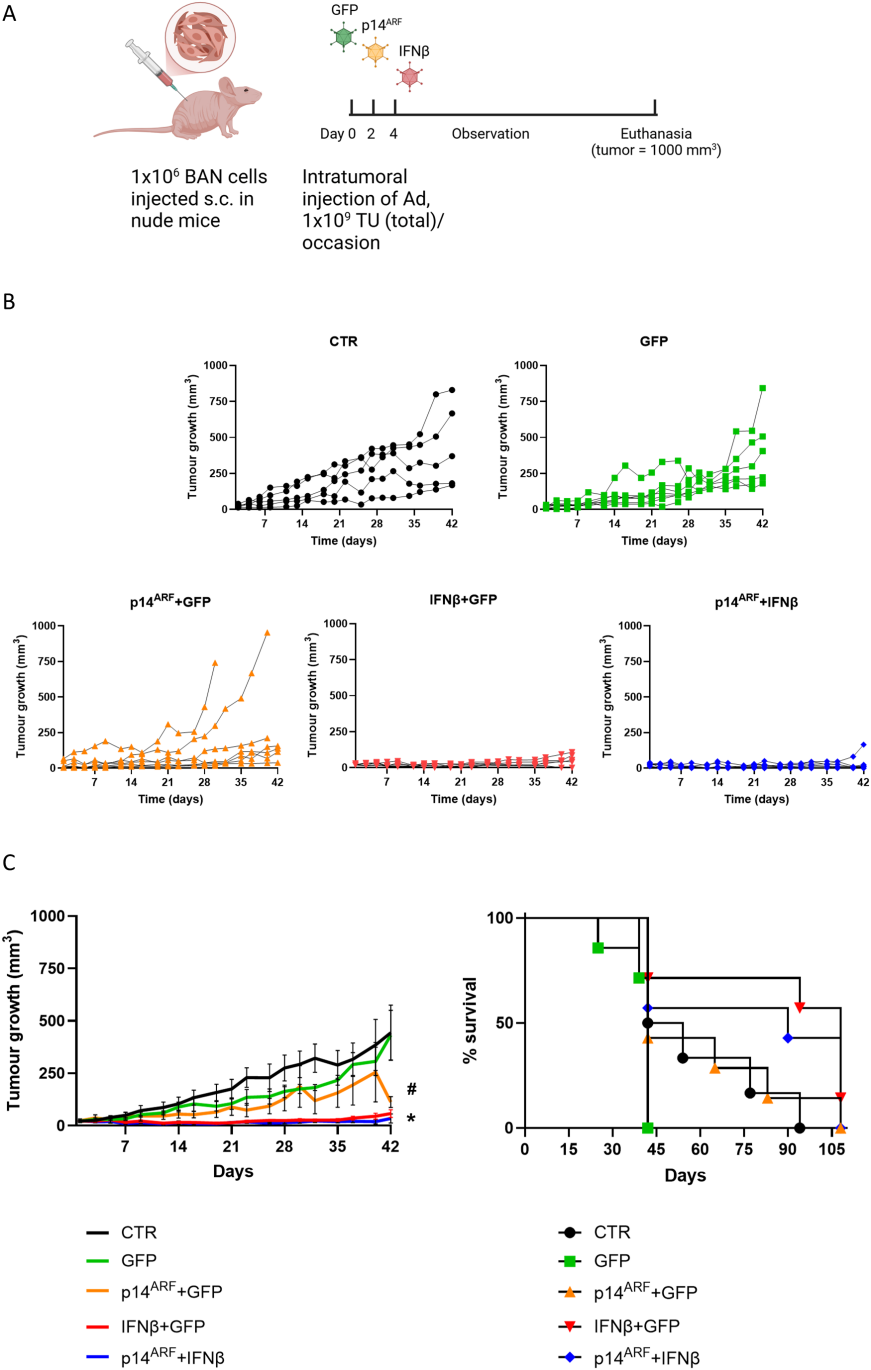
*In situ* gene therapy of BAN tumors in nude mice. **(A)** treatment is represented schematically. Note that a total of 1x10^9^ TU was used for each application and day 0 represents the first treatment. When necessary, the treatment was supplemented with the GFP vector to maintain consistent vector and gene dosage. **(B)** tumor volume of individual animals up to day 42 post treatment. **(C)** average tumor volume (+/s SEM for each experimental group) and Kaplan-Meier plots. ^#^p<0.05, p14^ARF^+GFP vs. CTR; *p<0.05, IFNβ+GFP vs. CTR or GFP; p14^ARF^+IFNβ vs. CTR or GFP. One way ANOVA, repeated measures, with Sidak’s multiple comparison test.

Next, we performed *in situ* gene therapy where the BAN cell line was injected s.c. in nude mice, the tumors were treated with adenovirus, and progression was observed (**Figure 6A**). As seen in **Figures 6B, C**, near total inhibition of tumor progression was seen up to day 42 post-treatment in the groups treated with IFNβ alone or in combination with p14^ARF^. p14^ARF^ also inhibited tumor progression in 4/6 animals. The PBS and GFP control tumors progressed steadily up to day 42. The Kaplan-Meier survival curve (**Figure 6C**) also reveals a greater number of surviving animals (tumors ≤1000 mm^3^) on day 107 in the groups treated with IFNβ alone or in combination with p14^ARF^.

## Discussion

We have isolated the tumorigenic BAN and TIG cell lines from canine oral melanomas. Each carries wild-type p53 and is susceptible to transduction with RGD-modified adenoviral vectors. We consider BAN and TIG to be transformed rather than merely immortalized, as they bypassed G1 arrest under serum starvation and readily formed tumors in nude mice. The cell lines were susceptible to transduction using adenoviral vectors of interest to our laboratory. Moreover, the transduction of p14^ARF^ or IFNβ induced cell death associated with the release of immunogenic cell death (ICD) markers *in vitro* and significantly inhibited tumor progression in an *in situ* gene therapy model.

Although neither cell line produced visible melanin, the BAN cell line was positive for the melanoma marker S100. This observation is consistent with previous reports indicating that melanin production in canine melanoma cell lines can cease after extended passages, as described by Inoue et al. (20). In contrast, the TIG cell line was negative for S100, Melan-A, HMB45, and tyrosinase. Previous studies have shown that progression from clinical stage I to IV is associated with loss of melan-A and tyrosinase expression in 14% of human cases (21, 22) and that a similar finding has been seen in cultured human cells (23). Nevertheless, we focused on the BAN cell line as a model for gene therapy in canine oral melanoma, as it consistently continued expressing at least one melanoma-associated marker.

*In vitro* and *in vivo* assays using established cell lines are a mainstay in biology, including the study of cancer. Canine cell lines are particularly compelling since findings from their use may benefit the understanding and treatment of cancer in both veterinary and human cases (2–4). Our own interest involves cancer gene therapy where we have developed a novel approach that, at least in mouse and human cell lines, induces immunogenic cell death (13, 15). As part of our efforts to conduct translational preclinical studies, we aim to evaluate the performance of our gene therapy approach in dogs. The first step involves the establishment of cell lines, followed by assessment of their response to gene transfer both *in vitro* and in a xenograft model of *in situ* gene therapy, as demonstrated in the present study. Both models are limited with respect to demonstrating immune activation. For this reason, we aim to apply our gene transfer approach in spontaneous cases of oral melanoma in companion dogs. This way, better investigating the immunological responses in a clinically relevant setting.

Since our gene therapy strategy uses adenovirus as a gene transfer vehicle and targets the p53 pathway, it is essential that the newly established cells be permissive to adenoviral transduction and we must determine the status of *TP53*. As demonstrated here, both newly established cell lines are permissive to transduction utilizing adenoviral vectors containing a modified knob protein. The insertion of an RGD tripeptide in the knob confers tropism mediated by integrins instead of the coxsackie-adenovirus receptor, thus broadening the spectrum of cells that may be transduced (24). We have not tested native Ad5 vectors in these cell lines since our own work relies on the RGD modified vectors. We have not yet tested canine adenoviruses, which have been studied for both human and veterinary application due to their lack of reliance on the coxsackie-adenovirus receptor and to avoid the issue of pre-existing antibodies that neutralize Ad5 (25).

The *TP53* gene appears to be wild-type in the newly isolated cell lines. For BAN and TIG, sequencing of exons 4 through 8 of *TP53* did not reveal any alterations as compared to the known sequence of canine *TP53*. As in human melanoma, alteration of *TP53* in canine melanoma is a rare event (26, 27). Nevertheless, mutation of *TP53* in either species tends to be concentrated in DNA binding domain encode by exons 5 through 8 (28, 29). Isolation of canine melanoma cell lines have been previously reported, including with extensive characterization of the p53 pathway, where 4/5 cell lines retained functional p53 that could be activated by inhibition of MDM2 or DNA damaging agents (30).

Functional evidence that *TP53* is wild type in the newly isolated cell lines was seen in several assays. Treatment of the cell lines with doxorubicin or Nutlin-3 was correlated with elevated p53 protein levels. In addition, upregulation of known p53 transcriptional targets was also observed upon drug treatment. Reporter gene assays showed that the p53-responsive promoter was activated upon drug treatment, whereas the constitutive CMV promoter was either not affected or activated to a lesser extent, consistent with our previous experience (31). A recent study has shown that gene expression driven by the CMV promoter is altered during cellular stress (32), a finding that may explain, in part, the results seen in **Figure 4** where drug treatment led to slight increases in CMV promoter activity. Overall, here we provide molecular and mechanistic evidence that the p53 protein in the BAN and TIG cell lines is functional.

The newly constructed adenoviral vectors encoding canine p14^ARF^ and IFNβ were used to transduce the BAN cell line, resulting in reliable transgene expression, induction of cell death, immunogenic cell death (ICD) markers release, and activation of key components of the p53 and type I IFN pathways. Consistent with our previous findings, IFNβ, either alone or in combination with p14ARF, effectively elicited antitumor responses. In our previous studies we have also found that human p14^ARF^ was sufficient to induce ICD in human cutaneous melanoma cell lines (17). In this study, we have also observed that canine p14^ARF^ was sufficient to induce immunogenic markers, such as the release of HMGB1 and activation of TRAIL.

Ad5 vectors have been studied in canine models, including companion dogs. Ad5 has been used to deliver canine factor VIII (FVIII) in hemophilic dogs by i.v. injection. Expression lasted only 5–10 days and was associated with liver toxicity, development of anti-FVIII antibodies and a transient drop in platelets (33). In another study, a high-capacity vector based on Ad5, which carries no viral genes, was used to deliver the canine FVIII gene i.v. in a hemophilic dog, showing only short-term transgene expression, partial correction of blood clotting time without the production of anti-FVIII antibodies, hepatotoxicity or hematologic alterations, suggesting that attenuation of adenoviral components may be necessary to avoid adverse events in dogs (34). While these studies show the problems and promise of using Ad5, they do not address the fact that Ad vectors do not provide long-term expression as needed for the treatment of genetic diseases.

Ad5 vectors are best suited for interventions that require only short-term expression of the transgene, such as for vaccines or cancer gene therapy, and these vectors have been evaluated in veterinary cancers. For example, Candolfi et al. have developed a gene therapy approach for the treatment of glioblastoma where non-replicating Ad5 encodes either thymidine kinase (TK) or Flt3L. In a pre-clinical test, they injected the combination of vectors in the brain of healthy dogs and observed increased immune infiltration at the site of treatment, as anticipated, but no adverse effects (35). Examination of transgene expression in the canine brain was confirmed when using a high-capacity Ad, leading the authors suggest that such vectors are an excellent choice for testing in canine glioblastoma, the ideal preclinical animal model. Ad5 has also been tested in 32 cases of canine melanoma, including oral tumors. Here the human CD40 ligand (CD40L) cDNA was encoded in a non-replicating Ad5 vector and delivered i.t. up to six times. This resulted in T and B lymphocyte infiltration, 7 complete responses and 5 partial responses. No serious adverse events or toxicity were noted, though transient increase in alanine amino transferase was seen in 5 dogs (36).

Use of vectors derived from Ad5 may encounter serious limitations, especially in human subjects. Systemic administration is generally not used since Ad5 is sequestered in the liver by Kupfer cells, thus the vector does not reach its target and also provokes humoral and cellular immunity. In addition, wild-type Ad5 causes respiratory infections that, while generally not problematic, do generate antibodies that neutralize *de novo* infections. Thus, a large portion of the human population carries pre-existing neutralizing antibodies (NAbs) against Ad5 (37). In human gene therapy trials, the impact of pre-existing or newly generated antibodies is not completely understood. In some instances, the NAbs limit the efficacy of virus administration, especially in repeated doses (38). However, there are also reports of benefit upon repeated administration (39) and complete responses even when NAbs are present (40). To circumvent the issue of NAbs, the virus may be modified or alternative vectors employed (37).

One option is canine adenovirus 2 (CAV-2), which is not sensitive to NAbs raised against human Ad5. Recombinant CAV-2 vectors have been developed, including non-replicating (E1/E3 deleted) and high-capacity (devoid of viral genes) vectors (41). In the veterinary setting, a vaccine against wild-type CAV-1, which causes infectious canine hepatitis, has been developed using modified live CAV-2. This vaccine relies on the development of neutralizing antibodies that have been shown to inhibit both CAV-1 and 2 (42). Thus, the use of CAV-2 vectors also has the potential to encounter limitations due to anti-viral immune responses.

While CAV-2 vectors have tropism for the upper respiratory tract, they also effectively transduce neurons, thus are being studied for gene therapy in the central and peripheral nervous system (41). Chimeric vectors between human and canine Ad have also been explored (43). CAV-2 oncolytic viruses (conditional replication due to E1A expression under the control of the osteocalcin promoter) have been developed (44) and their administration in normal dogs did not provoke toxicity, even when short-term immunosuppression was induced (45). Clearly, both Ad5 and CAV-2 vectors offer promise and reason for caution in their potential for veterinary and human gene therapy applications.

In conclusion, as demonstrated here, the newly established canine oral melanoma cell lines are well suited as a model for adenovirus-mediated gene transfer. Using the novel AdRGD-PG vectors, our combined p14ARF and IFNβ gene therapy approach induces robust cell death, triggers the release of key immunogenic cell death (ICD) markers, and inhibits tumor progression in a xenograft mouse model. However, the impact of these vectors on an immune-competent system could not be assessed in these preclinical models. Future studies will further characterize the cellular responses to treatment and will serve as a foundation for initial investigations in spontaneous cases of oral melanoma in companion dogs, enabling a better understanding of immune activation and providing a translational bridge for the development of therapies relevant to both veterinary and human melanoma.

## Data Availability

The raw data supporting the conclusions of this article will be made available by the authors, without undue reservation.
