# Supplementary material for: Adenovirus mediated gene therapy in cell lines derived from canine oral melanoma
Source: Front Immunol. 2026 May 28;17:1835389. doi: 10.3389/fimmu.2026.1835389 (PMC13254085; doi:10.3389/fimmu.2026.1835389)
Supplement: Supplementary file 1 [file DataSheet1.pdf]

Table S1: Characterization of s.c. tumors formed in nude mice using the cell lines established from canine melanomas.

Dog	Cell line (clone)	Morphology[a]	Melanin[a]	Tumorigenic[b]	Ki67	S100	Melanoma Coktail[c]	CD20	Cytokeratin Cocktail[d]	p53	PNL2
German shepherd	TIG (G2)	Fusiform	Absent	Yes	++++	-	-	-	-	-	-
Akita	BAN (C10)	Fusiform	Absent	Yes	++++	+	-	-	-	-	-

a: Observed in primary cell culture. b: Observed in nude mice after s.c. injection of $2x10^6$ cells. c: anti-melan-A, HMB45, tyrosinase. d: AE1, AE3. +, -, positive, negative detection. For Ki-67, relative intensity of the staining, as determined by the pathologist, is indicated.

Table S2: STR profiles[a] of the isolated cell lines.

Locus	BAN (Akita[b)])	TIG (German shepherd[b])
01 PEZ1	126	118
02 PEZ17	202	202/218
03 PEZ12	270/298	266/270
04 PEZ2	124/128	136
05 PEZ15	212/224	220
06 FHC2079	278	274
07 FHC2054	158	166/170
08 PEZ08	251	228/243
09 DogSRY	X/Y	X/Y
10 PEZ6	180/181	172/176
11 FHC2010	233	225/237

a, STR profiling performed by Cell Lines Service (Eppelheim, Germany).
b, 100% identitiy. Genetic confirmation of breed performed by Box4Pets (Taubaté, SP, Brazil)

Table S3: Oligonucleotides used in the RT-qPCR

Gene (canine)	Primer (5' – 3')
**p21 (CDKN1A)**	F: AAACGGCGGCAGACCAGCAT<br>R: GGTGTAGAAGGGGCCTGGGATGT
**MDM2**	F: GGGCCCCTTCGTGAGAATTG<br>R: GGTGTGGCTTTCTCAGGGATT
**HPRT1**	F: AGGACCCCTCGAAGTGTTGGC<br>R: GGGACTCCAGATGCTTCCAAACTCA
**IFNβ**	F: CATCCTCCAAACAACTCTCCT<br>R: TCCTGACACTCCAAACTGCT
**ISG15**	F: AAGCAGCAGATAGCCCTGAA<br>R: ACAGTTCTTCACCACCAGCA
**RIG-I**	F: GGAAGAACCAGAATGCCAGA<br>R: CCACAACCAGTAGGAGCACA
**TRAIL**	F: AAAGTGGCATCGCTTGTTTC<br>R: TGAGAACCTCGGTCGCTTAC
**GAPDH**	F: AACATCATCCCTGCTTCCAC<br>R: TGCCTGCTTCACTACCTTCTT
**Gene (human)**	**Primer (5' – 3')**
**p21 (CDKN1A)**	F: AGGAAGACCATGTGGACCTG<br>R: CGTTTGGAGTGGTAGAAATCTGTC
**MDM2**	F: AGGAGATTTGTTTGGCGTGC<br>R: TGAGTCCGATGATTCCTGCTG
**GAPDH**	F: TGCACCACCTGCTTAGC<br>R: GGCATGGACTGTGGTCATGAG

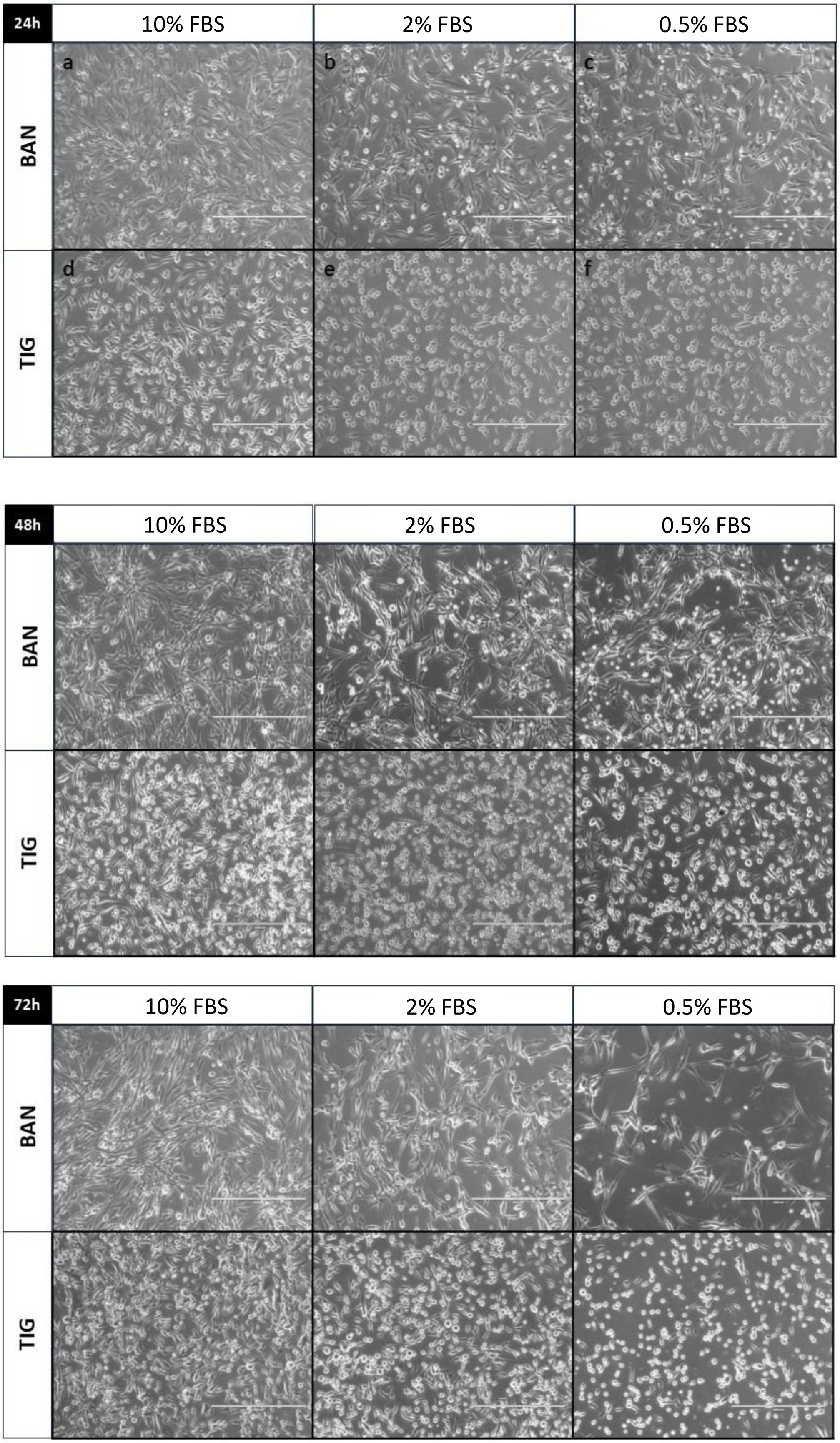

Figure S1 – Photomicrographs from serum starvation assay. Cells were treated as per Figure 1 of the main text. At the indicated time points, photomicrographs were captured (EVOS FL). Scale bar = 400 µm. Representative images are shown.

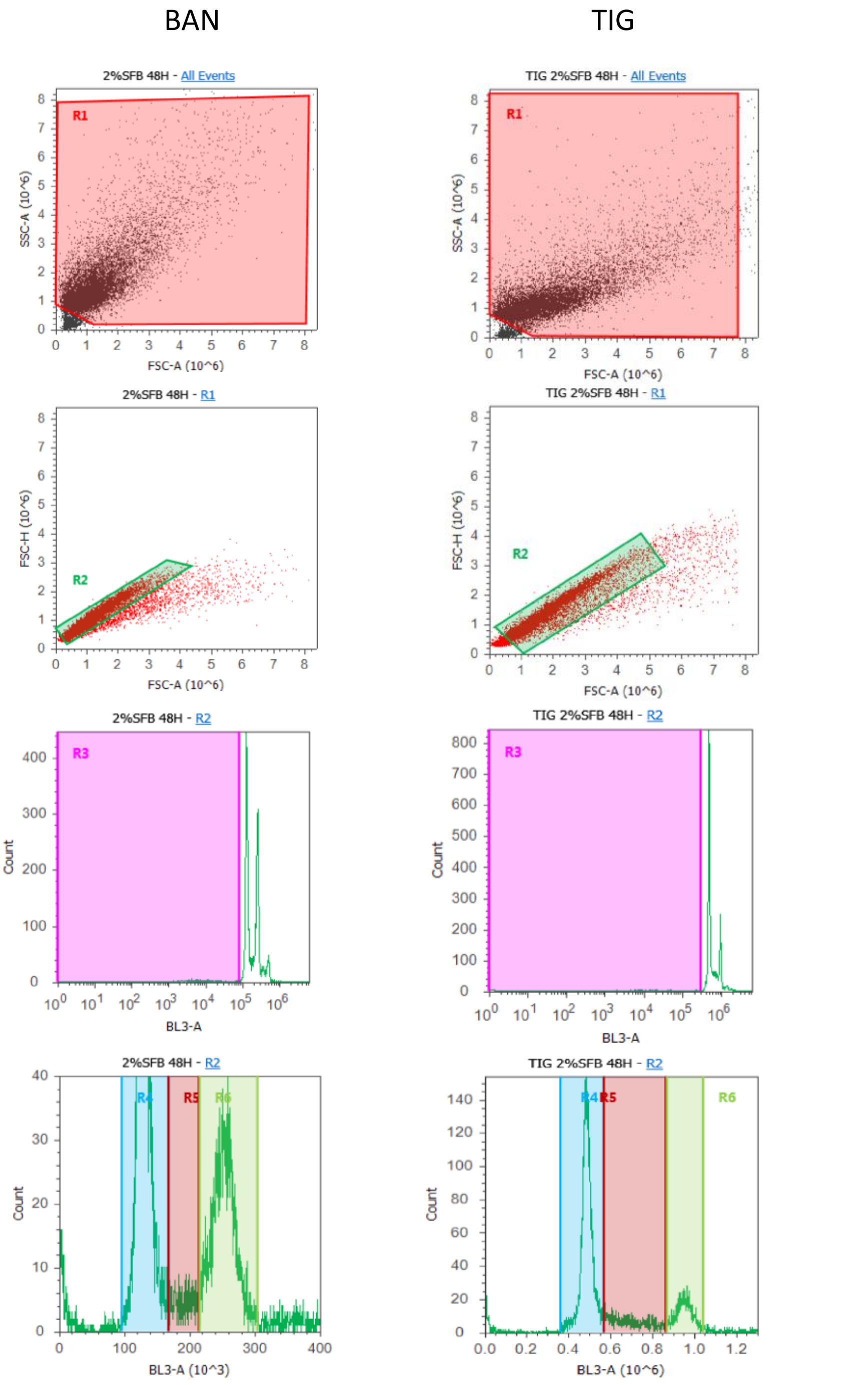

Parameters: **BL3-A vs Count**
Gate: **R2**
Experiment Name: **07-15-2022 11 40 AM**
Specimen Name: **BAN N1**
Sample Name: **2%SFB 48H**
Showing **14,128** of **14,128** events.

Name	Event Count	% Parent	% Total
All Events	14,128	100.000%	100.000%
R1	11,739	83.090%	83.090%
R2	10,200	86.890%	72.197%
R3	330	3.235%	2.336%

Experiment Name: **Restricao SFB N3 - Bianca - 08-12-2022 10 26 AM**
Specimen Name: **TIG N3**
Sample Name: **TIG 2%SFB 48H**
Showing **13,496** of **13,496** events.

Name	Event Count	% Parent	% Total
All Events	13,496	100.000%	100.000%
R1	11,821	87.589%	87.589%
R2	9,999	84.587%	74.089%
R3	324	3.240%	2.401%
R4	6,068	60.686%	44.961%
R5	1,461	14.611%	10.825%
R6	1,596	15.962%	11.826%

Figure S2: Example of the cytometry parameters used for determining the cell cycle distribution. Cells were treated as per Figure 1 of the main text. Shown here is a typical example of the cells cultivated in the presence of 2% FBS for 48 hours. For each cell line, the R1 population was further evaluated as single cells (R2), Sub-G1/Hypodiploid (R3), G1 (R4), S (R5) and G2/M (R6).

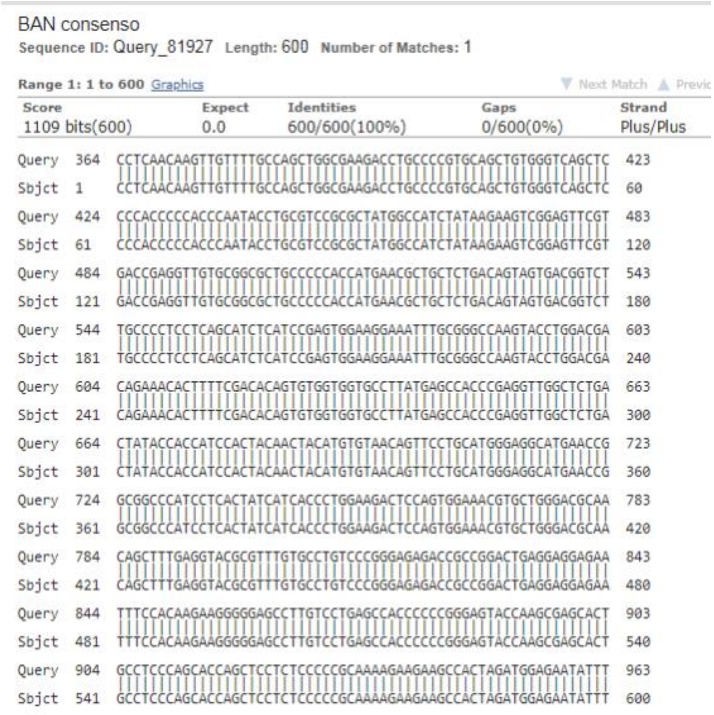

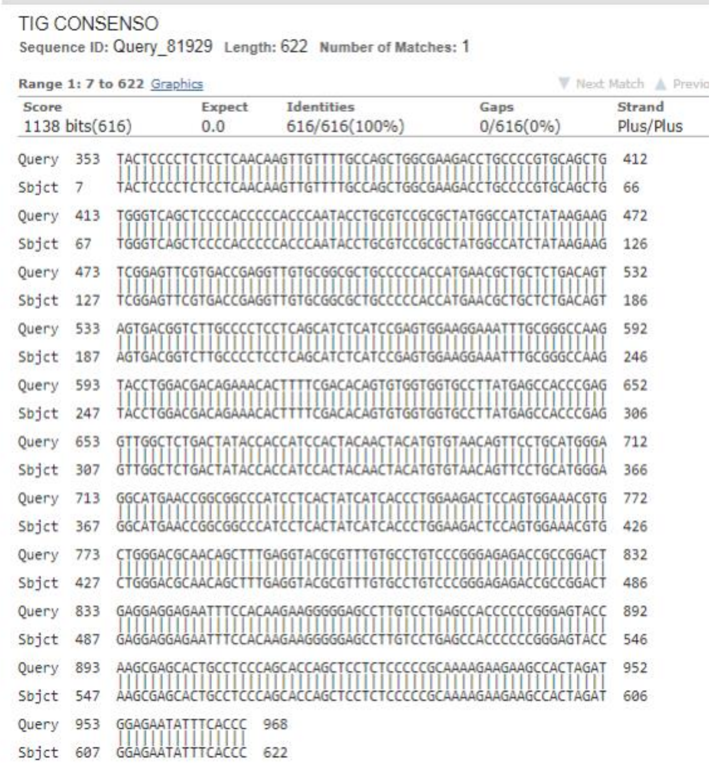

Figure S3: Global alignment of known *TP53* sequence with the sequence determined for the canine melanoma cell lines. The cDNA of exons 4 – 8 of the *TP53* gene obtained from the BAN and TIG canine melanoma cell lines was sequenced by the Sanger method. The sequences obtained were compared with the canine *TP53* (*Canis* lupus familiaris) from the NCBI database using the BLASTN 2.8.0 program, revealing 100% homology.

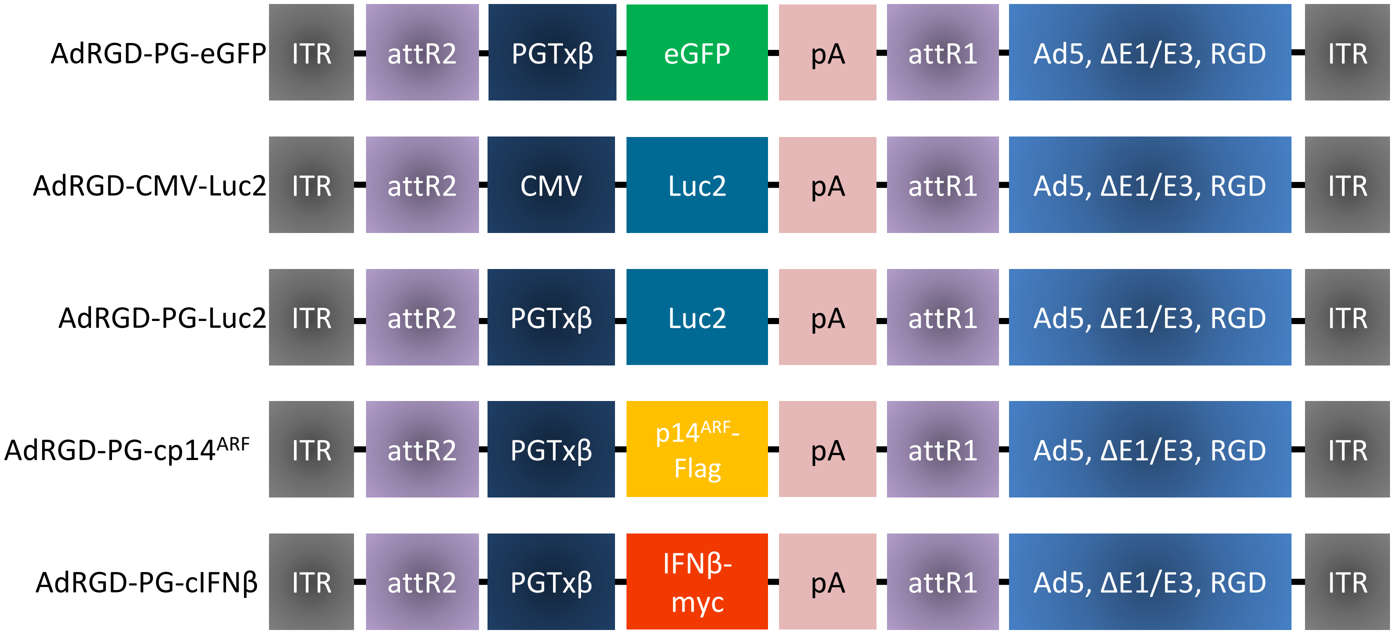

Figure S4. Schematic representation of the adenoviral vectors. ITR, inverted terminal repeat; attR2, attR1, site specific recombination sites; PGTxβ, chimeric p53 responsive promoter, where PG represents the p53 responsive element, Tx, minimal promoter, and β, intron from rabbit β-globin gene; eGFP, enhanced green fluorescent protein cDNA; Luc2, luciferase 2 cDNA; p14^{ARF}-Flag, canine p14^{ARF} cDNA fused with Flag tag; IFNβ-myc, canine IFNβ cDNA fused with myc tag; pA, polyadenylation site, Ad5, serotype 5 adenovirus backbone, ΔE1/E3, deletion of the E1 and E3 genes; RGD, tripeptide modification of the H1 loop of the knob protein.

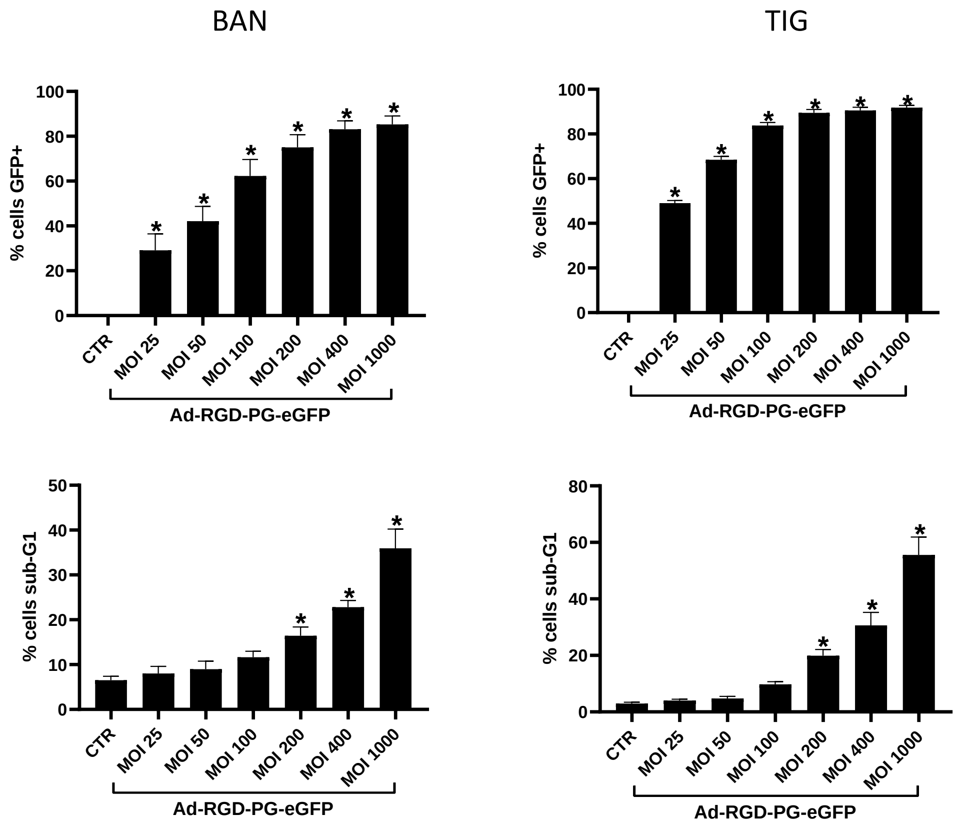

Figure S5: Determination of the transduction efficiency and toxicity. The BAN and TIG cell lines were transduced with AdRGD-PG-eGFP using increasing MOIs, as indicated. After 72h incubation, the cells were collected for detection of reporter gene activity and accumulation of sub-G1 cells.

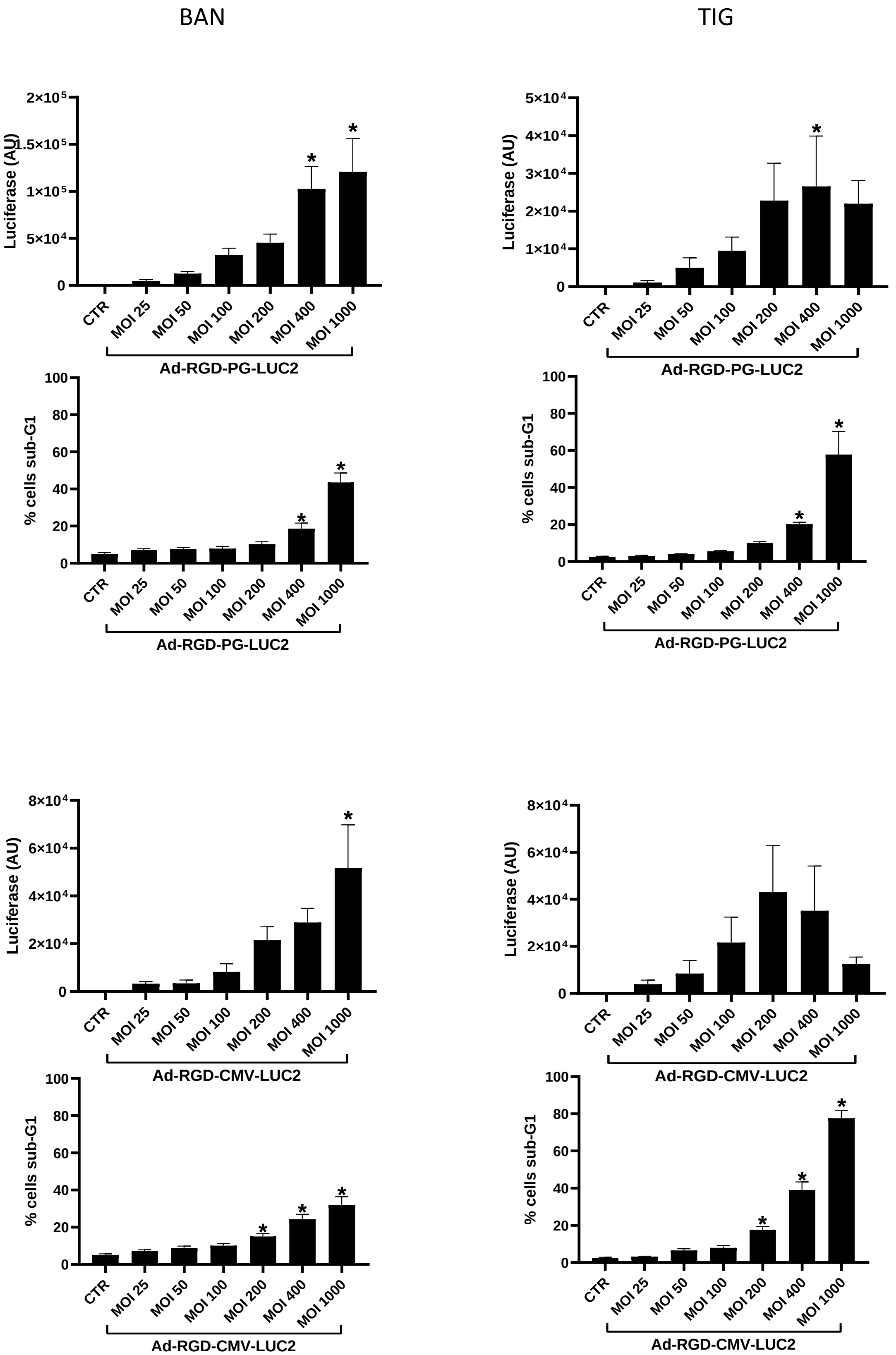

Figure S6: Determination of the transduction efficiency and toxicity. The BAN and TIG cell lines were transduced with AdRGD-PG-LUC2 or AdRGD-CMV-LUC2 using increasing MOIs, as indicated. After 72h incubation, the cells were collected for detection of reporter gene activity (normalized by protein content) and accumulation of sub-G1 cells. AU, arbitrary units. *, $p<0.05$ vs. CTR. One-way ANOVA with Dunnet post test.

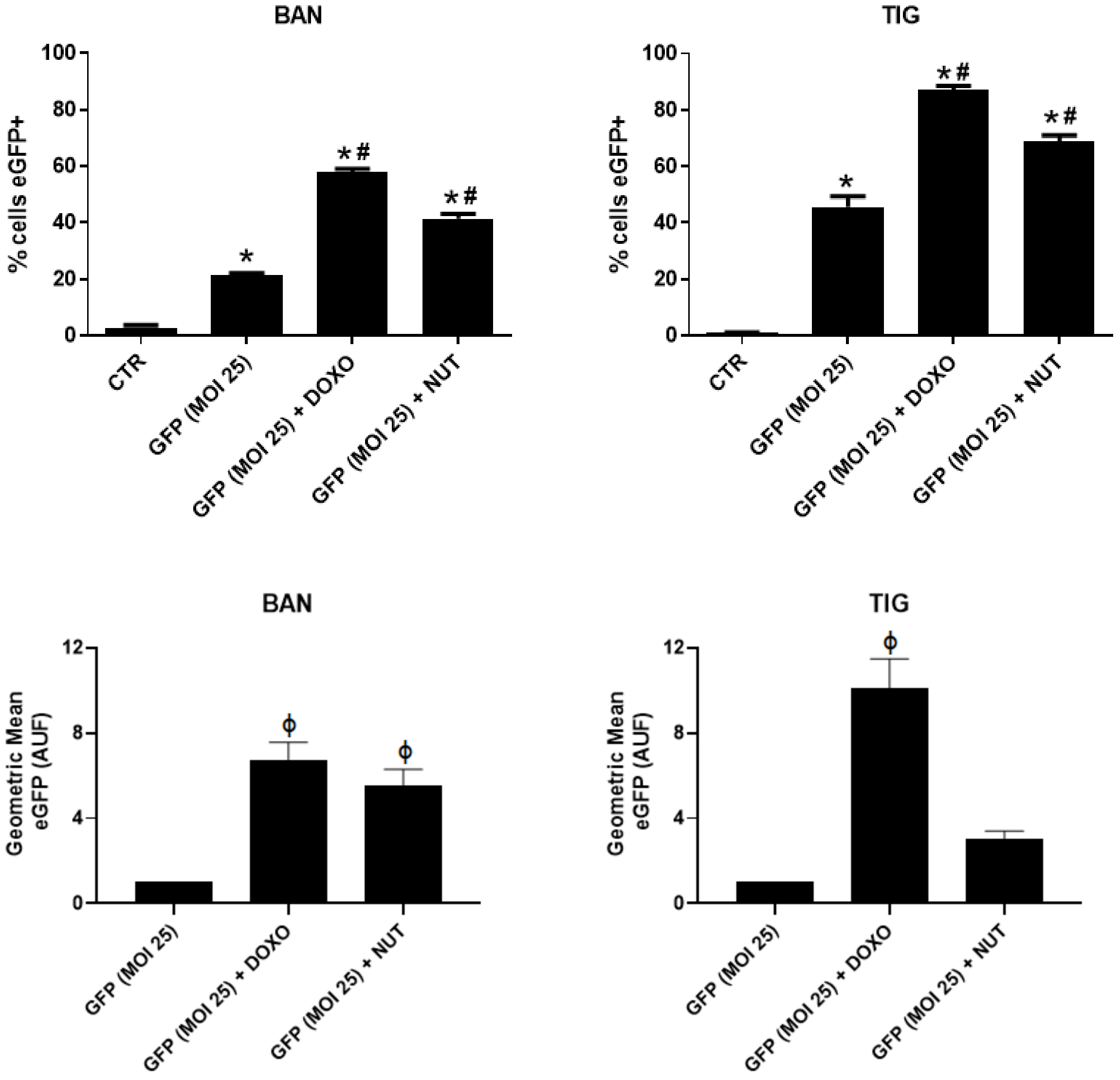

Figure S7: Reporter activity from the AdRGD-PG-eGFP vector was increased on treatment with drugs known to activate p53. The canine melanoma lines with no treatment (CTR) or the cells transduced with the AdRGD-PG-eGFP vector (GFP MOI 25) were cultivated for 24 hours before treatment with 0.1 µM doxorubicin (DOXO) or 10 µM Nutlin-3 (NUT), incubated for 48 hours then collected and analyzed by flow cytometry. The graphs on the upper panel represent the percentage of eGFP positive cells and on the bottom panel represent the geometric mean eGFP fluorescence intensity (AUF, arbitrary units of fluorescence). GFP condition normalized to CTR, then each DOXO and NUT normalized to GFP. Biological assays were performed on three independent occasions, each with technical replicates. One Way Anova with Tukey's post test. *, $p<0.05$ vs CTR and #, $p<0.05$ vs GFP (MOI 25). One Way Anova with Bonferroni's post test. φ, $p<0.05$ vs GFP (MOI 25).

A

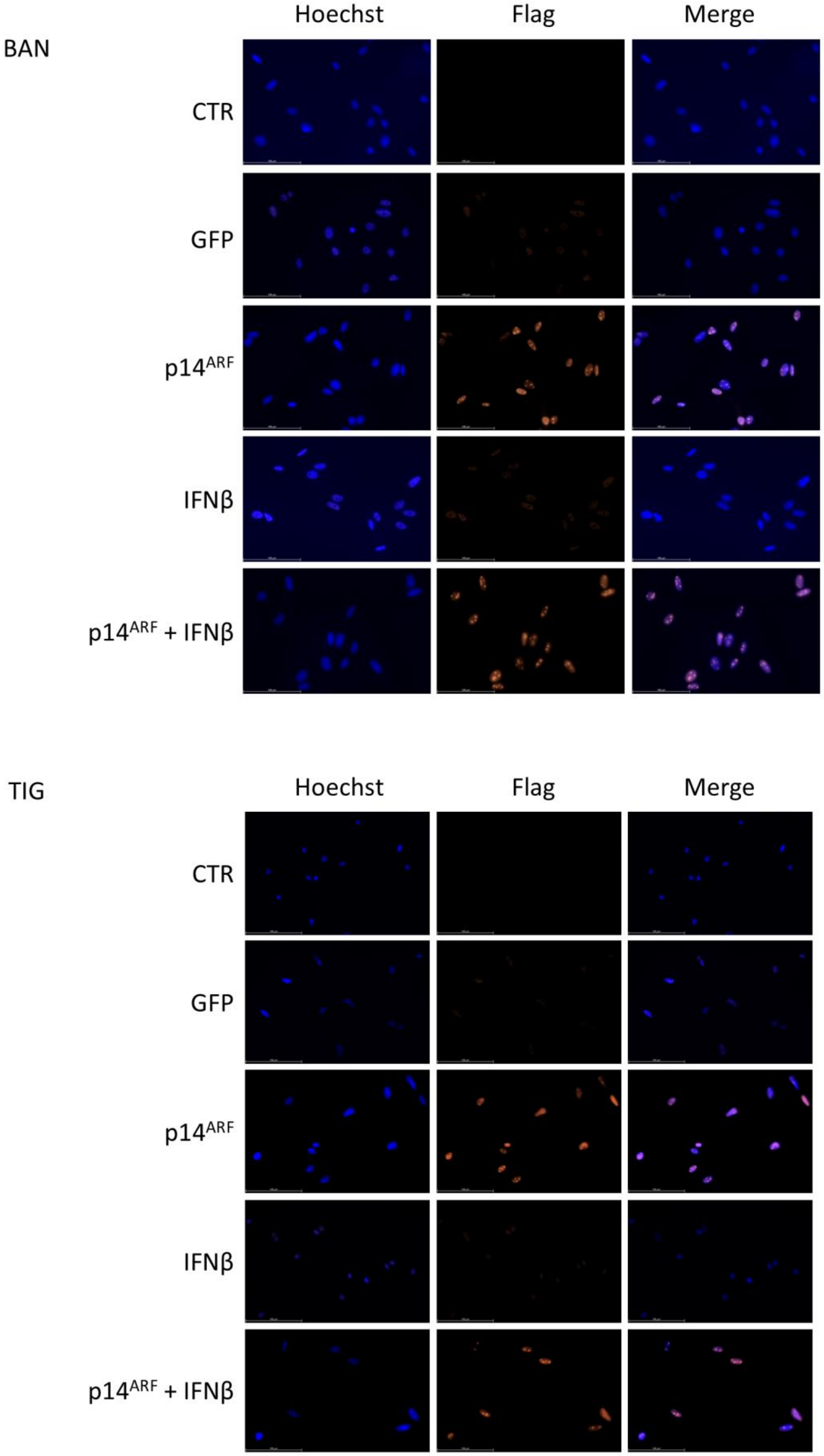
BAN
Hoechst
Flag
Merge
CTR
GFP
p14^{ARF}
IFNβ
p14^{ARF} + IFNβ
TIG
Hoechst
Flag
Merge
CTR
GFP
p14^{ARF}
IFNβ
p14^{ARF} + IFNβ

B

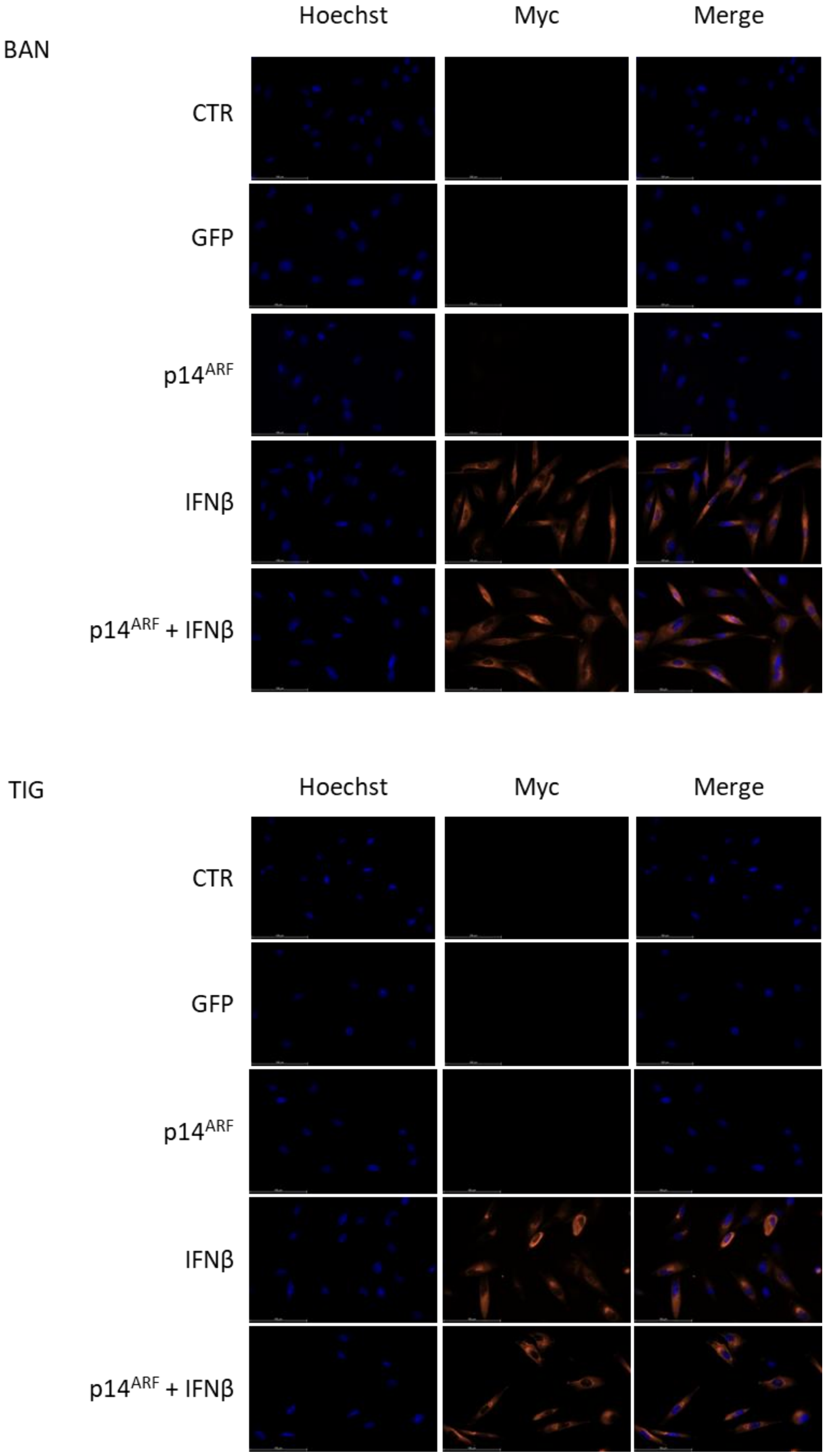
BAN
Hoechst
Myc
Merge
CTR
GFP
p14ARF
IFNβ
p14ARF + IFNβ
TIG
Hoechst
Myc
Merge
CTR
GFP
p14ARF
IFNβ
p14ARF + IFNβ

Figure S8: Detection of the exogenous p14ARF and IFNβ proteins in transduced cells by immunofluorescence. The canine melanoma lines with no treatment (CTR) or the cells transduced (MOI=200) with the AdRGD-PG-eGFP (GFP), AdRGD-PG-p14^{ARF} (p14^{ARF}) or AdRGD-PG-IFNβ vector. Detection was performed using a primary antibody specific for the tag and then staining with the secondary antibody conjugated with the fluorophore. Photomicrographs, all with 40x objective, were obtained using a Leica DMi8 fluorescence microscope. Size bar = 100 µ. **(A)** Detection of the flag-p14ARF fusion protein and **(B)** detection of the myc-IFNβ fusion protein. Note that in (B), the cells were treated with Brefeldin-A to promote intracellular accumulation of the myc-IFNβ fusion protein.

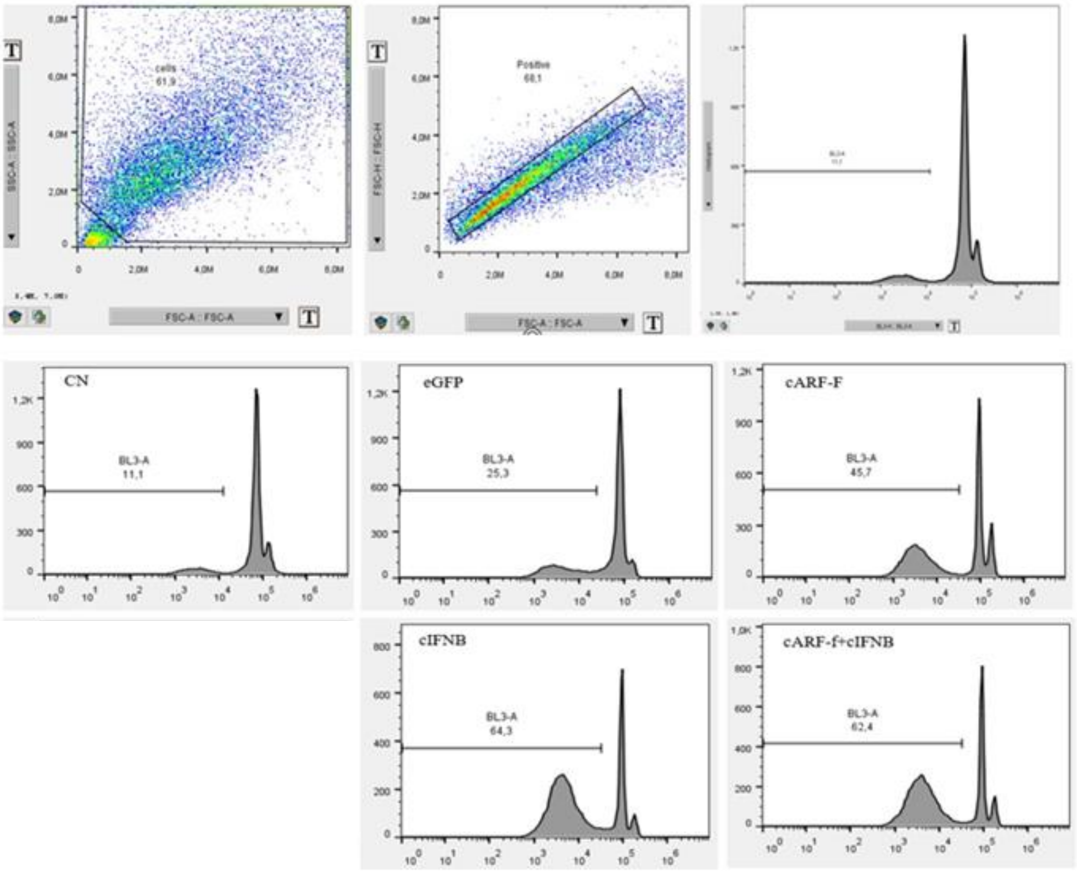

Figure S9: Gating strategy for the detection of the sub-G1 (hypodiploid) cell population. BAN was transduced as per Figure 5 of the main text, incubated for 72h, fixed and stained with PI before flow cytometry. A representative example of the non-transduced control (CN) or cells transduced with AdRGD-PG vectors expressing eGFP (G), p14^{ARF} (A) IFNβ (I), or the combination (I+A), is shown.

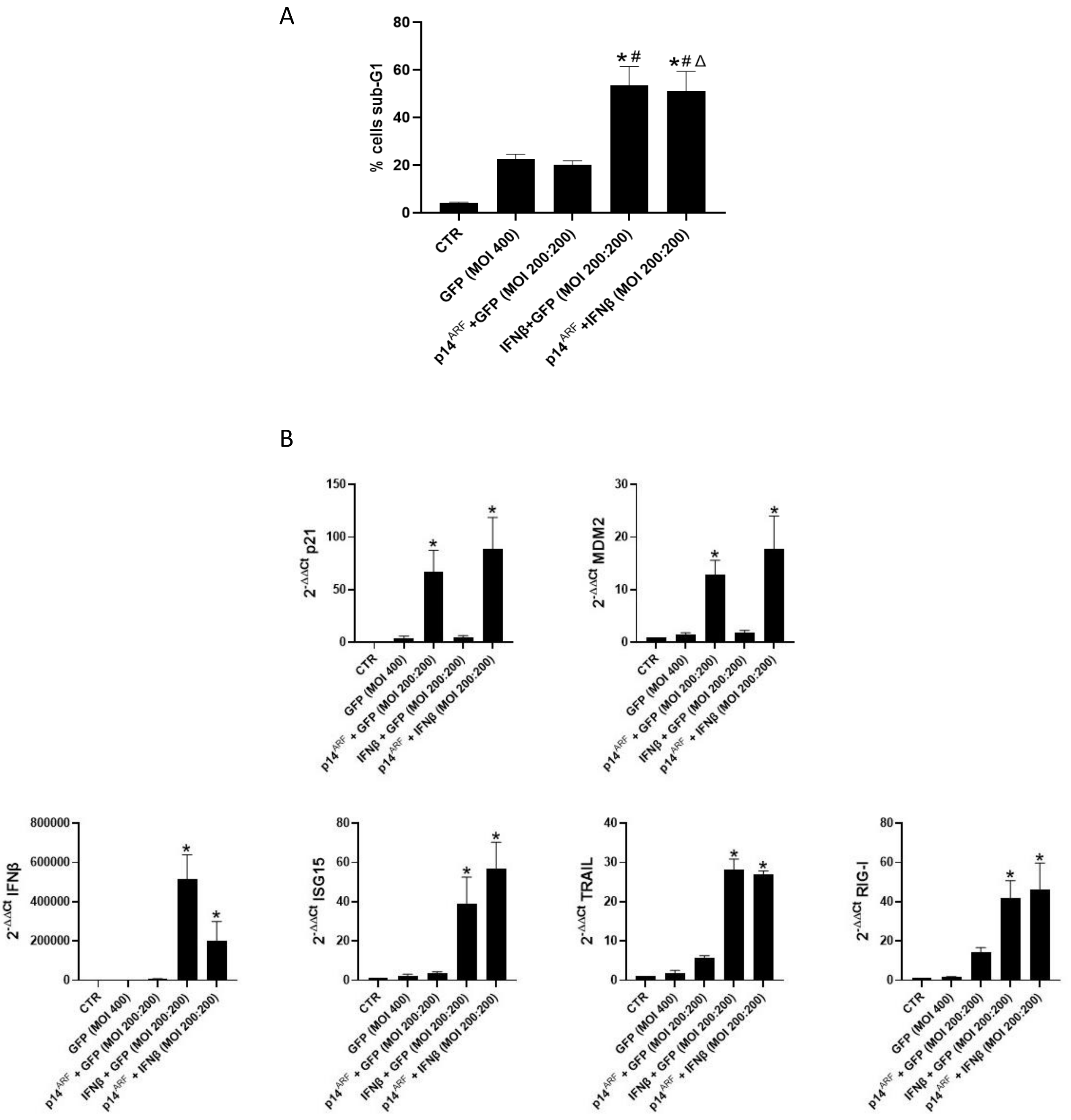

Figure S10: Response of TIG to *in vitro* treatment with p14ARF and IFNβ. Cells were transduced with consistent MOI (400) and gene dosage, as indicated. (**A**) Sub-G1 assay where cells were transduced in 12-well plates, incubated for 72 hours before harvesting, fixation, and staining with PI. N=3 biologic assays each performed with technical duplicates. *, $p<0.05$ vs CTR; #, $p<0.05$ vs. GFP; φ, $p<0.05$ vs $p14^{ARF}$+GFP and IFNβ+GFP. Ordinary One-way ANOVA, Bonferroni's post test. (**B**) RT-qPCR was performed using total RNA isolated from the cells 48 hs post transduction. At least 3 biological assays were performed. *, $p<0.05$; 2-way ANOVA, Tukey's post test.
